# Validation of deep-learning-based MRI-to-CT attenuation correction for striatal and extrastriatal [^123^I]I-FP-CIT SPECT measurement

**DOI:** 10.1016/j.nicl.2026.104020

**Published:** 2026-06-06

**Authors:** Sebastian Kalytta, Hendrik Theis, Kathrin Giehl, Merle C. Hoenig, Inés Mérida, Nicolas Costes, Adrian L. Asendorf, Manuel Reifegerst, Alexander Drzezga, Stéphane Prange, Thilo van Eimeren

**Affiliations:** a University of Cologne, Medical Facility and University Hospital of Cologne, Department of Nuclear Medicine, Cologne, Germany; b University of Cologne, Medical Facility and University Hospital of Cologne, Department of Neurology, Cologne, Germany; c Research Center Jülich, Institute for Neuroscience and Medicine (INM-2), Jülich, Germany; d CERMEP-Imagerie du Vivant, Bron, France; e German Center for Neurodegenerative Diseases, Bonn-, Cologne, Germany; f Univ Lyon, Lyon Neuroscience Research Center (CRNL), CNRS UMR 5292, INSERM U1028, Bron F-69675, France; g Hospices Civils de Lyon, Hôpital Neurologique Pierre Wertheimer, Service de Neurologie C, Centre Expert, Parkinson NS-PARK/FCRIN Network, Bron F-69500, France

**Keywords:** Deep learning, Attenuation correction, Quantitative analysis, [^123^I]I-FP-CIT, Ioflupane, SPECT

## Abstract

**Purpose:**

Attenuation correction is critical for accurate SPECT brain imaging and to quantify uptake ratios in neurological and psychiatric disorders. This prospective study aimed to validate the use of *deep-learning-based magnetic resonance to synthetic computed tomography* (DL-MRAC) attenuation correction by quantifying striatal and extrastriatal binding of [^123^I]I-FP-CIT to dopamine and serotonin transporters in Parkinson's disease patients.

**Methods:**

Synthetic CTs were generated from T1-weighted MRIs acquired in 12 Parkinson's disease patients using a validated 3D residual U-Net for attenuation correction of [^123^I]I-FP-CIT SPECT scans. We tested for equivalence of DL-MRAC versus CT-based attenuation correction (CTAC). We further compared uniform correction using Chang's method (UAC) and no attenuation correction (NAC) for regional analysis of specific binding ratios (SBRs) in striatal and extrastriatal areas. Data from the Parkinson's Progression Markers Initiative were used for external validation (*n* = 18).

**Results:**

As compared to CTAC, mean bias of DL-MRAC SBRs was −0.4% (95% confidence interval, CI -1.2 to 0.4) in the striatum and − 0.1% (95% CI, −0.8 to 0.6) in extrastriatal areas. UAC overestimated SBRs with mean bias ranging from 7.5% to 12.4%, whereas NAC underestimated SBRs with mean bias ranging from −6.5% to −24.0%, for striatal and extrastriatal binding estimates in all cohorts.

**Conclusion:**

DL-MRAC is a valid, radiation-free method for attenuation correction and quantification of [^123^I]I-FP-CIT binding to dopamine and serotonin transporters in subjects undergoing DaTSPECT examinations. It outperforms UAC and NAC and may therefore serve as a valuable alternative for patients for whom MRI is available and for data acquired on SPECT-only cameras.

## Highlights

• Attenuation correction is essential for [^123^I]I-FP-CIT SPECT measurements.
• A pretrained neural network generates accurate subject-specific synthetic CT from MRI data.
• Attenuation correction using deep-learning synthetic CT is equivalent to gold standard CT.
• Use of a pretrained network eliminates data collection, training, and radiation related to CT.

## 1 Introduction

Single-photon emission computed tomography (SPECT) using [^123^I]I-FP-CIT binding to the dopamine transporter is a well-established technique in the diagnostic assessment of parkinsonism (Morbelli et al., 2020). However, SPECT imaging is largely impacted by the attenuation and scatter of emitted photons, which results in physical deterioration of the signal with up to 50% decrease (Crespo et al., 2008; Schiebler et al., 2023) but can be mitigated by attenuation and scatter correction methods (Hashimoto et al., 1999). These methods are critical for the accuracy of quantitative SPECT imaging and its use as a diagnostic and prognostic biomarker in clinical and research settings. Accordingly, attenuation and scatter correction not only improves quantitative evaluation of [^123^I]I-FP-CIT in high-binding regions as the striatum and its correlation to clinical markers (Lapa et al., 2015), but also enables the detection of quantitative changes in extrastriatal regions and their evolution across time (Joling et al., 2019; Joling et al., 2017; Nicastro et al., 2020b). Notably, due to its affinity for serotonin transporters (Booij and Kemp, 2008), [^123^I]I-FP-CIT also enables the assessment of the raphe serotonergic system in the brainstem (Barber et al., 2018), whose projections are affected in Alzheimer's and Parkinson's disease (Ouchi et al., 2009; Prange et al., 2025; Prange et al., 2022). These anatomically small structures located deep inside the skull may be particularly prone to attenuation effects. Therefore, attenuation and scatter correction are critical to perform quantitative analysis of striatal and extrastriatal [^123^I]I-FP-CIT binding in healthy individuals and patients with psychiatric and neurodegenerative disorders.

Traditionally, Chang's method is used to perform calculated uniform attenuation correction (UAC) without employment of actual individual attenuation maps (Chang, 1978). Alternatively, an acquisition of a low-dose CT can be performed to enable non-uniform, subject-specific CT-based attenuation correction (CTAC). As such, CTAC improves the accuracy of quantitative SPECT imaging in comparison with UAC but entails additional radiation exposure to the patient (Inui et al., 2018; Ishii et al., 2012; Shibutani et al., 2017). Multiple radiation-free attenuation correction methods have been developed for PET and SPECT (Chen and Liu, 2023), including multi-atlas approaches (Burgos et al., 2014a; Mérida et al., 2017), MRI-based segmentation (Aasheim et al., 2015; Keereman et al., 2010), and deep learning methods, as used for brain-perfusion SPECT (Murata et al., 2021; Sakaguchi et al., 2021) or [^99m^Tc]Tc-TRODAT-1 SPECT (Du et al., 2023; Huang et al., 2025).

However, no study has yet tested the equivalence and performance of a non-uniform, radiation-free attenuation correction method for [^123^I]I-FP-CIT striatal and extrastriatal quantitative SPECT imaging. We addressed this issue by implementing a previously validated deep-learning algorithm to generate MRI-based synthetic CT (sCT) for attenuation correction (DL-MRAC) using the open-source, pretrained U-net model by Yaakub et al. (Yaakub et al., 2023) across a prospective validation cohort and two external validation cohorts from the Parkinson's Progression Marker Initiative (PPMI) database (Marek et al., 2011). We assessed the accuracy of attenuation maps obtained from sCTs by comparing them to ground truth CTs (gCTs) and aimed to test the equivalence of DL-MRAC in comparison to CTAC for quantitative [^123^I]I-FP-CIT SPECT imaging in cortical, subcortical, and brainstem regions for use in the clinical and research settings.

## 2 Materials and methods

#### 2.1.1 Participants

Twelve patients with Parkinson's disease were recruited from the University Hospital Cologne and participated in our prospective validation cohort designed for an equivalence test of DL-MRAC versus CTAC as detailed below (German Clinical Trials Register DRKS00032584). Participants were included if they had undergone [^123^I]I-FP-CIT SPECT at the Department of Nuclear Medicine at University Hospital Cologne for diagnostic purposes and agreed to additionally undergo a routine T1-weighted MRI and low-dose head CT. The external validation cohort included 18 subjects from the PPMI database that have undergone a [^123^I]I-FP-CIT SPECT combined with low-dose head CT and 3 T T1-weighted MRI (Nicastro et al., 2020b). Based on MRI, CT, and SPECT acquisition parameters, they were divided into two independent datasets (*external validation cohorts 1* and *2*; *n* = 9 each). Within each cohort, acquisition parameters were identical across all three imaging modalities (Table S1). Details about the datasets can be found in the Supplementary Methods.

#### 2.1.2 Study procedure

In our prospective validation cohort, all participants underwent [^123^I]I-FP-CIT SPECT using a Picker Prism3000 three-head SPECT system with low energy high resolution parallel-hole collimators and a low-dose head CT on a Siemens Biograph mCT Flow 128 Edge PET/CT scanner at the Department of Nuclear Medicine of the University Hospital Cologne. Three-dimensional anatomical T1-weighted sequences (MPRAGE) were acquired using a Siemens Magnetom Prisma 3 T scanner at the Research Center Juelich. Details on image acquisition and reconstruction parameters for all three modalities are provided in Table S1.

### 2.2 Synthetic CT generation and SPECT reconstruction

**Fig. 1:**
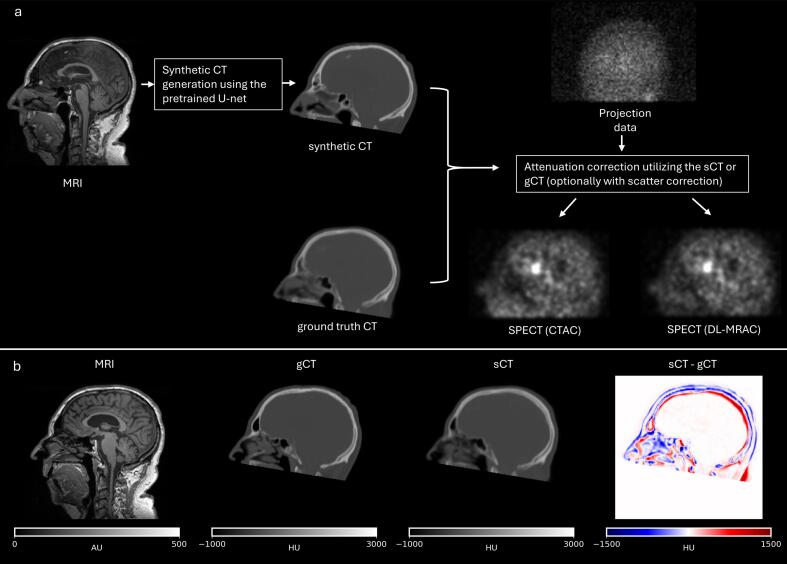
**S**ynthetic CT (sCT) generation for SPECT attenuation and scatter correction. **a** First, synthetic CT images (sCTs) were generated from the MRI data. Subsequently, the ground truth CT (gCT) and the sCTs were used for attenuation and scatter correction of the projection data. **b** MRI, gCT, sCT, and the corresponding difference image are shown for a representative subject from the PPMI database. HU: Hounsfield units; AU: arbitrary units. CTAC: ground truth CT-based attenuation correction. DL-MRAC: deep-learning MR-based attenuation correction.

Fig. 1 illustrates the pipeline for attenuation and scatter correction. MRIs were bias-field corrected and resampled to 1 mm isotropic voxel size. These MRIs were subsequently used as input to the pretrained model (3D residual U-Net) to generate the subject-specific sCT (Yaakub et al., 2023). Next, rigid registration aligned the ground truth CT (gCT) with the MRI via normalized mutual information using SPM12 (Wellcome Trust Centre for Neuroimaging, London, UK). For all three datasets (*prospective validation cohort*, *external validation cohort 1* and *2*), attenuation correction of the raw SPECT data was performed using gCT, sCT, and Chang's method (UAC) within Hermes Hybrid Recon (referred to as CTAC, DL-MRAC and UAC). Additionally, scatter and collimator correction were applied to all SPECT in the *prospective validation cohort* using implemented Hermes routines. Scatter correction was applied to the *external validation cohort 1* but could not be applied to *external validation cohort 2* due to missing radius of rotation. Collimator correction was not feasible for either *external validation cohort* due to undocumented collimator parameters (hole diameter and length). Additionally, all three datasets included non-attenuation/scatter-corrected (NAC) SPECT reconstructions. Further details on synthetic CT generation and SPECT reconstruction are provided in the Supplementary Methods.

### 2.3 Synthetic CT accuracy

For each subject, the MRI was non-linearly registered to the 6th-generation MNI152 T1 template provided in FSL, using ANTs and mutual information for SyNRA transformation (Avants et al., 2011). The same transformation was applied to both the gCT and the sCT. All analyses were performed within a head mask generated from the gCT images using Otsu thresholding (Otsu, 1975), followed by a 5 mm dilation and a closing operation to fill any holes. Dilation was performed to include a margin of air around the patient to account for potential errors in cases of minor misalignment (Huijben et al., 2024). Each generated sCT was compared to the subject's gCT by calculating the mean absolute error (MAE) in Hounsfield units (HU). The preprocessing pipeline (MRI bias field correction and field of view) was optimized to improve sCT accuracy (Fig. S1). The MAE was calculated across the head mask and separately for the bone window (>300 HU) using the following formula:

(1) $$\mathrm{MAE}=\frac{\sum_{i=1}^{N}\left|{\mathrm{sCT}}_{i}-{\mathrm{gCT}}_{i}\right|}{N}$$

Here, sCT_i_ and gCT_i_  refer to the voxel values (in HU) of the sCT and gCT, respectively, at voxel i, and N is the total number of voxels in the region of interest.

### 2.4 SPECT analysis

We included 71 regions of interest (ROIs) using the Harvard-Oxford cortical and subcortical structural atlases (48 cortical, 21 subcortical regions) and the dorsal and median raphe nucleus obtained from the Harvard Ascending Arousal Network Atlas (Edlow et al., 2012). The lateral occipital lobe inferior division was used as reference region for semiquantitative analysis using specific binding ratio (SBR). The 71 ROIs were aligned with the patient's MRI using non-rigid registration and the SPECT scans that were already co-registered with the MRI were resliced to the corresponding MRI using trilinear interpolation in SPM12. We calculated the relative error of the SBR for NAC, UAC and DL-MRAC relative to ground truth CTAC as follows:

(2) $$\mathrm{Relative error}\;\left(\%\right)=\frac{{\mathrm{SBR}}_{\mathrm{Method}}-{\mathrm{SBR}}_{\mathrm{gCT}}}{{\mathrm{SBR}}_{\mathrm{gCT}}}\times 100$$

In Eq. 2, SBR_Method_ was replaced by SBR_NAC_, SBR_UAC_, or SBR_DL-MRAC_, accordingly. The paired Wilcoxon signed-rank test was employed to assess the statistical significance of the bias in SBR. Bland-Altman analysis was performed to compare SBR across 70 regions (71 - the reference region) in the three cohorts.

To validate the use of the radiation-free DL-MRAC method using sCT in place of conventional CTAC for quantitative SPECT imaging, we designed a prospective validation cohort and assessed the equivalence of these two methods in both striatal and extrastriatal areas. As such, testing for equivalence relies on the null hypothesis that DL-MRAC SBRs were different from conventional CTAC SBRs, which eventually allows to demonstrate that sCT SBR are clinically equivalent to gCT SBR if the SBR differences are smaller than the priori defined equivalence margin, using the two one-sided *t*-test method for paired data (Wyatt et al., 2024). The equivalence margin thus represents the greatest SBR difference which would be deemed as clinically unimportant, therefore allowing to replace the reference method by the safer and simpler one, without impeding on the accuracy in a clinical or research setting. Our study is the first to directly test the equivalence of two such methods for attenuation correction for SPECT imaging, and no equivalence margin was previously reported. Therefore, we calculated the equivalence margin so that the difference of the uncertainties related to DL-MRAC and CTAC remained below 5%, considering the within-subject variability estimated from test-retest repeatability of [^123^I]I-FP-CIT SPECT. Accordingly, the only additional SBR uncertainty ∆AC between DL-MRAC and CTAC SBR was due to the uncertainty related to DL-MRAC, which was independent of all other uncertainties related to SPECT imaging (including reconstruction and measurement, which were contained in the CTAC uncertainty component), and therefore sums in squares following the propagation of uncertainty for independent variables as in Wyatt et al. (Wyatt et al., 2024):

(3) $$∆\mathrm{DL}\_{\mathrm{MRAC}}^{2}=∆{\mathrm{CTAC}}^{2}+∆{\mathrm{AC}}^{2}$$

We calculated the root mean squared error coefficient of variation of test-retest repeatability of [^123^I]I-FP-CIT binding in the striatum in Parkinson's disease patients (Booij et al., 1998), corresponding to ∆CTAC (6,53%). As such, the equivalence threshold ∆AC was 9,50%, resulting in overall uncertainty between DL-MRAC and CTAC below 5% by definition. To help contextualize, this equivalence margin corresponds to half of the difference between CTAC and UAC for striatal SBR observed in previous studies on Parkinson's disease patients (ranging from 20,19% to 23,5%) (Huang et al., 2025; Lapa et al., 2015). Accordingly, we calculated the sample size to test for equivalence in a prospective crossover validation cohort as in Julious et al. (Julious, 2004), using two one-sided paired *t*-tests (Walker and Nowacki, 2011) and assuming a null expected mean difference and no sequence effect. Bonferroni correction was used to test for equivalence in two ROIs (one striatal and one extrastriatal) with nominal *p* = 0.025, resulting in 12 patients for our prospective validation cohort with 80% power. We further performed a sensitivity analysis and tested the equivalence of DL-MRAC and CTAC so that the overall SBR uncertainty remained below 0.5% resulting in an equivalence threshold ∆AC of 2.6%.

In addition, voxel-wise SBR were calculated in MNI space for each method (NAC, UAC, DL-MRAC and CTAC) and used to depict the group-averaged difference maps as well as to perform voxel-wise paired statistical testing complementary to the regional analyses. Specifically, voxel-wise difference maps in MNI space were computed for NAC, UAC, and DL-MRAC relative to CTAC for each patient. Paired voxel-wise testing was then performed using FSL's *randomise* toolbox with threshold-free cluster enhancement and family-wise error correction at *p* < 0.05. In addition, voxel-wise correlations (joint histograms and linear regression) and Bland-Altman analyses were performed across all three cohorts.

To assess within-subject reproducibility of the proposed DL-MRAC approach, a supplementary test-retest analysis was performed in a subset of subjects from *external validation cohort 2*. Four subjects underwent repeat MRI acquisitions 2–4 years after baseline. Synthetic CT images were generated from the repeat MRIs and used for attenuation correction of the same SPECT data originally reconstructed with the baseline MRI. SBR were subsequently compared between baseline- and repeat MRI-based reconstructions across 70 brain regions by calculating regional percentage differences.

## 3 Results

### 3.1 Synthetic CT generation and accuracy

Construction of sCT followed by DL-MRAC was successfully performed in all patients and compared to other attenuation correction methods. In the *prospective validation cohort*, MAE of sCTs was 125.2 ± 12.0 HU within the head mask and 264.0 ± 33.1 HU within the bone window compared to gCTs. In the *external validation cohort*, MAE within the head mask was 115.3 ± 6.7 HU for *cohort 1* and 126.2 ± 6.6 HU for *cohort 2,* and 265.2 ± 15.8 HU for *cohort 1* and 258.6 ± 17.6 for *cohort 2* within the bone window.

### 3.2 Regional analysis

Evaluation of the SBR from [^123^I]I-FP-CIT reconstructed with DL-MRAC compared to CTAC demonstrated clinical equivalence within the predefined equivalence margins for both striatal (*p* < 0.001) and extrastriatal regions (p < 0.001) for the *prospective validation cohort*. The mean bias of DL-MRAC SBR was −0.4% (95% CI, −1.2 to 0.4) in the striatum and − 0.1% (95% CI, −0.8 to 0.6) in extrastriatal areas, demonstrating the equivalence of DL-MRAC and CTAC within the 2.6% equivalence margin defined in the sensitivity analysis. For the *external validation cohort 1,* the mean bias of DL-MRAC SBR was −1.7% (95% CI, −2.4 to −1.0) in the striatum and − 1.5% (95% CI, −2.2 to −0.8) in extrastriatal areas. For the *external validation cohort 2,* the mean bias of DL-MRAC SBR was −0.6% (95% CI, −1.8 to 0.6) in the striatum and − 0.2% (95% CI, −1.3 to 0.8) in extrastriatal areas. Increased mean bias for striatal and extrastriatal SBR was observed for UAC, ranging from 7.5% to 12.4%, and for NAC, ranging from −6.5% to −24.0%, across the prospective and external validation cohorts.

**Fig. 2:**
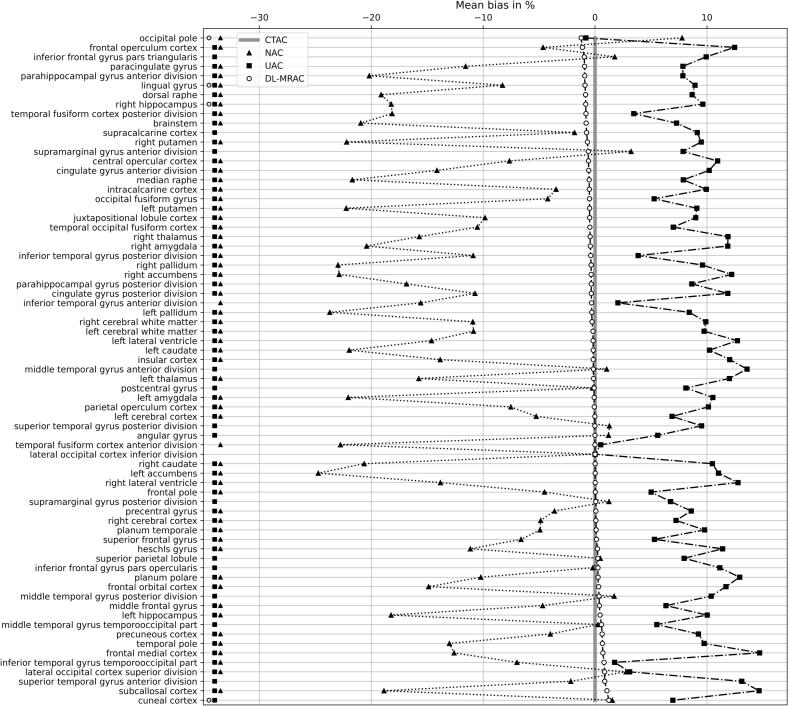
Prospective validation cohort. Mean SBR bias per ROI and method, sorted by DL-MRAC bias from most negative to most positive. Symbols represent SBR approaches: NAC (triangle), UAC (square), DL-MRAC (circle). Bias in % is represented on the x-axis. Paired Wilcoxon signed rank test at the regional level (*p* < 0.05). On the left side of the figure symbols indicate that the regional SBR of the respective method was significantly different from the ground truth SBR (CTAC).

**Fig. 3:**
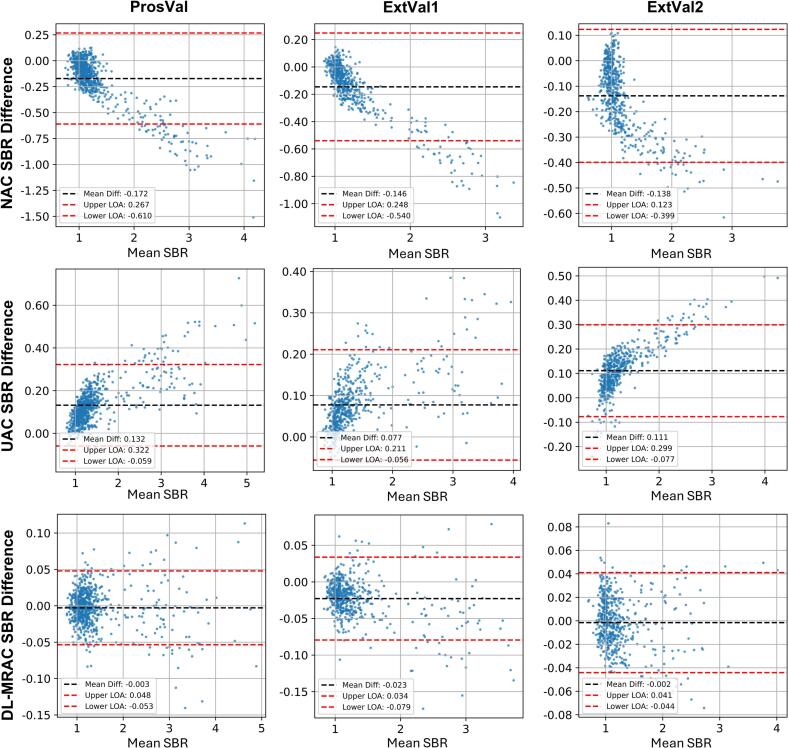
Bland-Altman plots of SBR for NAC, UAC, and DL-MRAC across 70 ROIs for *prospective validation cohort* (ProsVal), *external validation cohort 1* (ExtVal1), and *external validation cohort 2* (ExtVal2). The x-axis represents the mean SBR of CTAC and the respective method (NAC, UAC, and DL-MRAC), while the y-axis shows the difference between CTAC and the corresponding method. The mean differences and limits of agreement (LOA), defined as ±1.96 standard deviations, are depicted. The x-axis and y-axis scaling is adjusted to each method.

Fig. 2 presents the in-depth regional analysis of the mean SBR bias across the 70 ROIs using NAC, UAC, and DL-MRAC with reference to CTAC. Mean bias ranged from −1.2% to +1.2% for DL-MRAC across all ROIs, with significant bias in 4 of 70 regions (Table 1). UAC resulted in larger errors, ranging from −0.8% to +14.7%, with a tendency for overestimation and significant bias in 67 regions. NAC showed a bias ranging from −24.7% to +7.8% with a tendency toward underestimation and a significant bias in 56 regions. Similar results were obtained for *external validation cohorts 1* and *2* (Fig. S2, Fig. S3, Table S2, Table S3). In the Bland-Altman analysis, DL-MRAC had lower mean difference and narrower distribution, with lower standard deviations compared to UAC or NAC in all three cohorts (Fig. 3). UAC caused an overestimation of the SBR, which was more pronounced in higher-intensity ROIs. In contrast, NAC caused an underestimation, which was more pronounced in higher-intensity ROIs.

**Table 1:** Prospective validation cohort. Mean bias and standard deviation of SBR (%) for the NAC, UAC, and DL-MRAC methods compared to CTAC for the striatum, pallidum, raphe nucleus and brainstem.

	NAC	UAC	DL-MRAC
**Caudate L**	−21.97 ± 4.59 ⁎	10.22 ± 3.63 ⁎	−0.16 ± 1.12
**Caudate R**	−20.63 ± 3.98 ⁎	10.48 ± 3.51 ⁎	0.01 ± 1.39
**Putamen L**	−22.24 ± 3.95 ⁎	9.09 ± 3.54 ⁎	−0.49 ± 1.66
**Putamen R**	−22.21 ± 3.79 ⁎	9.49 ± 3.48 ⁎	−0.68 ± 1.60
**Pallidum L**	−23.71 ± 4.15 ⁎	8.42 ± 3.67 ⁎	−0.29 ± 1.92
**Pallidum R**	−22.97 ± 4.32 ⁎	9.60 ± 3.62 ⁎	−0.35 ± 1.46
**Dorsal Raphe**	−19.12 ± 4.23 ⁎	8.68 ± 6.10 ⁎	−0.85 ± 2.04
**Median Raphe**	−21.70 ± 4.57 ⁎	7.88 ± 5.51 ⁎	−0.53 ± 2.46
**Brainstem**	−20.94 ± 3.66 ⁎	7.29 ± 4.53 ⁎	−0.80 ± 1.57

⁎ p < 0.05 Wilcoxon signed rank test. R, right; L, left.

In the supplementary test-retest analysis, four subjects from the *external validation cohort 2* with repeat MRI acquisitions acquired 2–4 years after baseline were evaluated. Synthetic CTs generated from the repeat MRIs were used for attenuation correction of the same baseline SPECT data, and regional percentage differences in SBR were assessed across 70 brain regions. Across all 280 region-wise comparisons, the largest negative difference was −2.4% and the largest positive difference was +2.7%, while most regional differences were below 1% (Fig. S5, Table S4). Although the limited sample size precludes definitive conclusions regarding test-retest performance, the findings provide supportive evidence for good within-subject repeatability.

### 3.3 Voxel-wise analysis

**Fig. 4:**
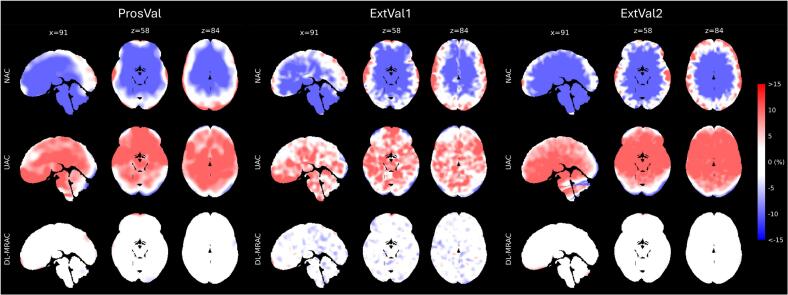
Mean voxel-wise bias (in %) of NAC, UAC and DL-MRAC versus CTAC for the *prospective validation cohort* (ProsVal), *external validation cohort 1* (ExtVal1) and *external validation cohort 2* (ExtVal2). Z = 58 goes through the midbrain, z = 84 through the caput of the caudate. The color scale was thresholded at −15% and + 15% to improve the visualization of local errors. Voxels with bias less than the 2.6% equivalence margin were color-coded in white.

**Fig. 5:**
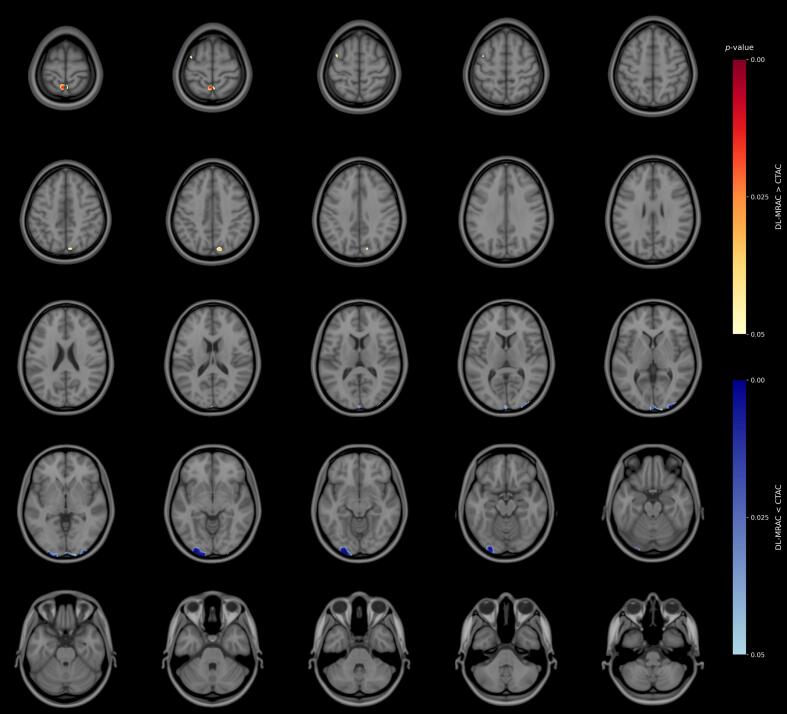
Voxel-wise paired testing of DL-MRAC versus CTAC in the prospective validation cohort. Significant clusters of overestimation (DL-MRAC > CTAC) are shown in red, whereas significant clusters of underestimation (DL-MRAC < CTAC) are shown in blue. Clusters not reaching statistical significance (*p* < 0.05) are not displayed.

Mean voxel-wise difference maps of the SBR for NAC, UAC and DL-MRAC versus CTAC are depicted in Fig. 4 for the *prospective validation cohort* and *external validation cohort 1* and *2*. DL-MRAC showed the lowest voxel-wise bias across all three cohorts, within the equivalence margin for DL-MRAC across the whole brain*. External validation cohort 1* showed regions of underestimation, consistent with the trend for underestimation observed in Fig. 3 and Fig. S2. UAC yielded for all three datasets an overestimation of the central regions and an underestimation in the periphery of the occipital, and cerebellar regions. NAC resulted in an opposite pattern with a strong underestimation of the central regions of the brain and an overestimation of peripheral areas of the brain. The results of the voxel-wise paired testing for DL-MRAC are shown in Fig. 5. For DL-MRAC, almost no significant clusters of underestimation or overestimation were observed, with only small focal clusters in the right occipital pole and superior parietal region in the prospective cohort, indicating minimal residual differences relative to CTAC. In contrast, UAC exhibited pronounced clusters of overestimation in central regions across all three cohorts (Fig. S7), whereas NAC demonstrated the opposite pattern, with central underestimation (Fig. S8). These spatial patterns are consistent with the mean bias findings from Fig. 4. The results of the voxel-wise paired testing for UAC and NAC in the prospective validation cohort, together with the corresponding results for DL-MRAC, UAC, and NAC in external validation cohorts 1 and 2 showing similar patterns, are provided in the Supplementary Material (Fig. S9-S14).

### 3.4 Correlation analysis

#### 3.4.1 Voxel-wise joint histogram

**Fig. 6:**
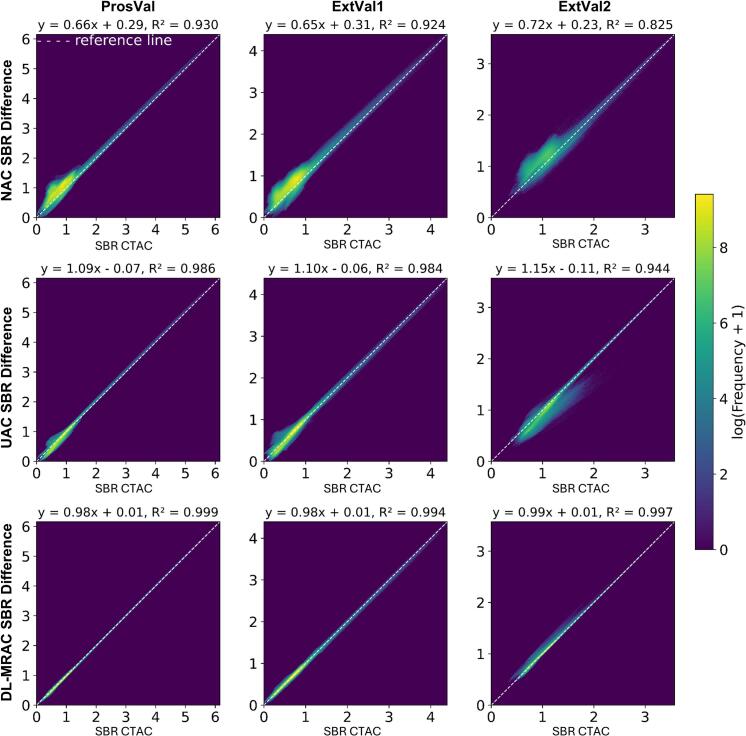
Voxel-wise joint histogram analysis of the SBR comparing CTAC with NAC, UAC, and DL-MRAC for *prospective validation cohort* (ProsVal), *external validation cohort 1* (ExtVal1) and *external validation cohort 2* (ExtVal2). The result of the linear regression analysis, along with the corresponding coefficient of determination (R^2^), is shown at the top of each plot. The logarithmic representation was chosen to better visualize less frequent SBR. The x-axis and y-axis scaling is adjusted to each method.

Fig. 6 presents the results of the voxel-wise joint histogram analysis for the three cohorts and three reconstruction methods. Among all three cohorts, the distribution was narrowest and most aligned with the identity line (reference line) for DL-MRAC. This was followed by UAC, while NAC performed the worst. The coefficient of determination resulting from the linear regression analysis was highest for the DL-MRAC method across all three cohorts, followed by UAC, with NAC performing the lowest.

## 4 Discussion

In this study, we demonstrated the equivalence of a radiation-free, deep learning-based approach for attenuation correction of [^123^I]I-FP-CIT SPECT in striatal and extrastriatal regions versus the reference CTAC method. We further validated its use for quantitative imaging in clinical and research settings based on three validation cohorts. Our results demonstrated that DL-MRAC has high agreement with CTAC at the regional and voxel-wise level and significantly outperformed UAC and NAC, thus opening the way for large scale and reliable quantitative [^123^I]I-FP-CIT SPECT imaging.

One advantage of a pretrained MRI-to-CT neural network is that it can be used universally for any tracer, provided that the synthetic CTs are sufficiently accurate. Cranial MRI is typically obtained as part of the diagnostic work-up in patients with Parkinson's disease and may subsequently serve as input for DL-MRAC (Berardelli et al., 2013). We showed that DL-MRAC improves the quantitative accuracy of [^123^I]I-FP-CIT SPECT imaging in both striatal and extrastriatal regions compared to UAC and NAC, enabling reliable assessments across multiple brain areas without the need for an actual CT scan. Using DL-MRAC, [^123^I]I-FP-CIT striatal SBRs could therefore be used as a biomarker, in particular to assess the progression of Parkinson's disease in longitudinal studies (Dzialas et al., 2025), its correlation with motor severity scores (Lapa et al., 2015) and motor reserve (Dzialas et al., 2025), but also to investigate the extrastriatal, non-dopaminergic binding to the serotonergic system (Barber et al., 2018; Joling et al., 2019; Nicastro et al., 2020b), and contribute to the diagnostic workup of parkinsonian disorders by enhancing the accuracy and reliability of extrastriatal signal evaluation (Joling et al., 2017; Nicastro et al., 2020a).

We found that bias of DL-MRAC SBR was very low in comparison to CTAC, with low variability for regional and voxel-wise analysis, whereas greater bias was observed for NAC and UAC. Consistent with previous findings, NAC tended to underestimate SBR, while UAC tended to overestimate it, with high inter-regional variability (Huang et al., 2025; Lange et al., 2014; Lapa et al., 2015). In particular, UAC overestimated SBR in centrally located structures such as the striatum, in line with earlier reports (Huang et al., 2025; Ishii et al., 2012; Lange et al., 2014; Lapa et al., 2015). Conversely, UAC underestimated SBR in the posterior brain regions in all three cohorts, a pattern that has also been reported previously (Ishii et al., 2012).

The results of the Bland-Altman plots are consistent with the findings of Du et al., who compared UAC and NAC with deep learning-based attenuation correction for dopamine transporter SPECT using [^99m^Tc]Tc-TRODAT-1 (Du et al., 2023). Their study also reported a general underestimation in NAC, which was more pronounced in higher-intensity regions. In contrast, UAC led to overestimation, following the same trend at higher intensities. These findings align with our results but contrast with those of Murata et al., who observed the opposite pattern, reporting an underestimation with UAC and an overestimation with NAC (Murata et al., 2021). However, it is important to note that Murata et al. evaluated brain-perfusion SPECT, where high-intensity regions are mostly located at the outer boundaries of the brain and tend to be underestimated by UAC, as signified by our results.

Overall, DL-MRAC for [^123^I]I-FP-CIT SPECT imaging represents a low-bias and highly reliable attenuation correction method, as has been demonstrated for PET and other SPECT tracers (Chen and Liu, 2023).

Several limitations need to be considered when interpreting the current results. A limitation of this study is that the datasets did not include patients with major structural abnormalities, such as tumors, hemorrhage, severe atrophy, or intracranial metal implants, limiting the generalizability of our findings to these populations. Only one patient presented with marked ventricular enlargement. In this case, the synthetic CT demonstrated slight hyperdense boundary artifacts at the interface between cerebrospinal fluid and brain parenchyma. These effects did not result in increased bias in the attenuation-corrected SPECT images compared with the remaining subjects. Across the three validation cohorts, the method demonstrated accurate results overall, however, due to the limited availability of cases with structural abnormalities in MRI or CT imaging, its applicability to such conditions remains insufficiently validated. Another limitation is that we did not account for attenuation of components such as the headrest, which contributes to attenuation in SPECT images. In combined SPECT/CT scanners, these components are accounted for during attenuation correction. We removed the headrest in the ground truth CTs of the prospective and external validation cohorts because these CTs were acquired on scanners that used different headrests from those employed during SPECT acquisition. Its inclusion could have adversely affected the quality of the SPECT images (Iwao et al., 2023). Furthermore, UAC, DL-MRAC, and CTAC all neglected headrest attenuation, warranting the validity of the direct comparison of these methods. However, in settings where SPECT/CT scanners are used, the CT scan can image the headrest, and it may be beneficial to integrate the image of the headrest into the sCT to account for its contribution to attenuation and scatter. This issue does not arise for PET/MR scanners with integrated hardware-based μ-maps in the reconstruction software (Mérida et al., 2017). Another limitation is that the CT scanners and tube voltages employed in the neural network's training dataset differ from those used in our validation cohorts. The CT scanners used for training the network had tube voltages of 140 and 100 kVp (Yaakub et al., 2023), while our CT scanner operated at a tube voltage of 120 kVp. This was also the case in the external validation cohorts 1 and 2 (110 kVp and 130 kVp, respectively). Although Hounsfield Units are normalized to water, there is a dependence between HU and tube voltage, as well as interscanner variations in measured Hounsfield Units (Sande et al., 2010). Another limitation stems from the differences in MR sequences and scanners employed for training the neural network compared to our own MR sequences and those used in the external validation cohorts. Although all the datasets consisted of T1-weighted images, there were variations in parameters such as repetition time, echo time, and flip angle. These differences may introduce inaccuracies in the construction of sCTs, as the input images deviate from the neural network's training set (Yaakub et al., 2023). Notably, scatter correction introduced additional noise, appearing as irregular speckles in the SBR difference maps. These artifacts disappeared when images were reconstructed using gCT and sCT with attenuation correction but without scatter correction (Fig. S4). A possible explanation is that correction methods dependent on an attenuation map may introduce additional errors if the attenuation map itself contains inaccuracies. These inaccuracies can alter the deflection of scattered photons in the Monte Carlo simulation, ultimately leading to the irregular pattern observed in the SBR bias image (Burgos et al., 2014b).

In conclusion, this study demonstrated that radiation-free, MRI-based attenuation correction using a pretrained model produces accurate SBR estimates which are clinically equivalent to CTAC for use in the clinics and research. Moreover, DL-MRAC outperformed UAC and NAC in three different cohorts with differences in SPECT and MR acquisition parameters, highlighting the strengths of an open-source pretrained U-net model, representing an efficient, portable, universal and reliable method to generate sCT for DL-MRAC without the need to retrain with new data. Overall, we showed that DL-MRAC is a valid method to replace CTAC for quantitative [^123^I]I-FP-CIT SPECT imaging in both striatal and extrastriatal regions, which emphasizes its role as a biomarker in psychiatric and neurodegenerative disorders and its use in multicentric studies.

## CRediT authorship contribution statement

**Sebastian Kalytta:** Writing – original draft, Visualization, Validation, Software, Methodology, Investigation, Formal analysis. **Hendrik Theis:** Writing – review & editing, Investigation. **Kathrin Giehl:** Writing – review & editing, Project administration. **Merle C. Hoenig:** Writing – review & editing, Project administration. **Inés Mérida:** Writing – review & editing, Software, Methodology. **Nicolas Costes:** Writing – review & editing. **Adrian L. Asendorf:** Writing – review & editing, Data curation. **Manuel Reifegerst:** Writing – review & editing, Software. **Alexander Drzezga:** Writing – review & editing, Resources. **Stéphane Prange:** Writing – review & editing, Validation, Supervision, Methodology, Formal analysis, Conceptualization. **Thilo van Eimeren:** Writing – review & editing, Validation, Supervision, Resources, Methodology, Funding acquisition, Conceptualization.

## Ethics approval and consent to participate

The study was approved by the Ethics Committee of the University Hospital Cologne (Date: 26/01/2023, No: 22-1214_1) and conducted in accordance with the standards of the Declaration of Helsinki and the national radiation protection authorities (“Bundesamt für Strahlenschutz”). Written informed consent was obtained from all individual participants included in the study.

## Funding

This project was supported by the German Research Foundation (DFG, Deutsche Forschungsgemeinschaft): project ID 431549029 - CRC 1451, C03. H.T. was supported by the Cologne Clinician Scientist Program (CCSP)/Faculty of Medicine/University of Cologne, as funded by the German Research Foundation (DFG, Deutsche Forschungsgemeinschaft): project No. 413543196. SP was supported by a research fellowship from the Alexander von Humboldt foundation.

## Declaration of competing interest

The authors declare that they have no known competing financial interests or personal relationships that could have appeared to influence the work reported in this paper.

## Data Availability

The data supporting this study’s findings are findable in the CRC1451 data registry (https://www.crc1451.uni-koeln.de/), and reasonable requests can be addressed to the corresponding author.
