# Supplementary material for: Validation of deep-learning-based MRI-to-CT attenuation correction for striatal and extrastriatal [123I]I-FP-CIT SPECT measurement
Source: Neuroimage Clin. 2026 Jun 6;51:104020. doi: 10.1016/j.nicl.2026.104020 (PMC13273211; doi:10.1016/j.nicl.2026.104020)
Supplement: Supplementary file 1 — Supplementary material [file mmc1.docx]

**Validation of deep-learning-based MRI-to-CT attenuation correction for striatal and extrastriatal**

**[^123^I]I-FP-CIT SPECT measurement**

Sebastian Kalytta ^a^, Hendrik Theis ^a,b^, Kathrin Giehl ^a,c^, Merle C. Hoenig ^a,c^, Inés Mérida ^d^, Nicolas Costes ^d^, Adrian L. Asendorf ^a^, Manuel Reifegerst ^a^, Alexander Drzezga ^a,c,e^, Stéphane Prange ^a,f,g,1^, Thilo van Eimeren ^a,b,1,*^

**Author affiliations:**

^a^ University of Cologne, Medical Facility and University Hospital of Cologne, Department of Nuclear Medicine, Cologne, Germany

^b^ University of Cologne, Medical Facility and University Hospital of Cologne, Department of Neurology, Cologne, Germany

^c^ Research Center Jülich, Institute for Neuroscience and Medicine (INM-2), Jülich, Germany

^d^ CERMEP-Imagerie du Vivant, Bron, France

^e^ German Center for Neurodegenerative Diseases, Bonn-Cologne, Germany

^f^ Univ Lyon, Lyon Neuroscience Research Center (CRNL), CNRS UMR 5292, INSERM U1028, Bron F- 69675, France

^g^ Hospices Civils de Lyon, Hôpital Neurologique Pierre Wertheimer, Service de Neurologie C, Centre Expert

Parkinson NS-PARK/FCRIN network, Bron F-69500, France

^1^ These authors contributed equally to this work.

* Corresponding author:

Thilo van Eimeren, MD, FEAN

University Hospital of Cologne

Department of Nuclear Medicine,

Kerpener Str. 62, 50937 Cologne, Germany

research office: +49 221 478 7570

clinical office: +49 221 478 4007

mail: [thilo.van-eimeren@uk-koeln.de](mailto:thilo.van-eimeren@uk-koeln.de)

# Supplementary Methods

## Acquisition parameters

**Table S1** Acquisition parameters of the three datasets prospective validation cohort (ProsVal), external validation cohort 1 and 2 (ExtVal1, ExtVal2)

| **Cohort** | | **ProsVal** | **ExtVal1** | **ExtVal2** |
| --- | --- | --- | --- | --- |
| **Number participants** | | 12 | 9 | 9 |
| **DaT-SPECT**  **parameters** | **Voxel size (mm)** | 3.047 isotropic | 3.296 isotropic | 2.572 isotropic |
|  | **Matrix size** | 128x128x128 | 128x128x128 | 128x128x128 |
|  | **Collimator** | Parallel hole | Parallel hole | Parallel hole |
|  | **AC** | Yes | Yes | Yes |
|  | **SC** | Yes | Yes | Not possible |
|  | **CC** | Yes | Not possible | Not possible |
| **MRI parameters** | **Voxel size (mm)** | 0.9x0.898x0x898 | 0.977x0.977x1.0 | 1.0x1.0x1.0 |
|  | **Matrix size** | 192x256x256 | 256x256x176 | 240x256x176 |
|  | **TE (ms)** | 2.32 | 2.48 | 2.98 |
|  | **TR (ms)** | 2300 | 1900 | 2300 |
|  | **TI (ms)** | 900 | 900 | 900 |
|  | **FA (°)** | 8 | 9 | 9 |
| **CT parameters** | **Voxel size (mm)** | 0.977x0.977x3 | 0.586x0x586x1.5 | 0.586x0.586x1.5 |
|  | **Matrix size** | 512x512x75 | 512x512x109 | 512x512x110 |
|  | **Tube voltage** | 120 kVp | 110 kVp | 130 kVp |

AC: attenuation correction, SC: scatter correction, CC: collimator correction, TE: echo time, TR: repetition time, TI: inversion time, FA: flip angle

## External validation cohorts

Imaging data for *external validation cohort 1* and *2* was obtained from the Parkinson's Progression Markers Initiative (PPMI) database (<https://www.ppmi-info.org/>). Eighteen complete datasets were identified, each containing CT scans, T1-weighted MRIs, and raw [^123^I]I-FP-CIT SPECT suitable for reconstruction using the Hermes Hybrid Recon software (HERMES Medical Solutions, Stockholm, Sweden). When multiple MRIs and SPECTs were available, the baseline MRI and SPECT were selected for analysis. For some patients, two CT scans reconstructed with different kernels (H30S and H19S) were available in the PPMI database. In these cases, the CT reconstructed with the H19S kernel was chosen. The 18 datasets were divided into two groups (*external validation cohort 1* and *2*), each containing nine datasets, based on the acquisition parameters of the SPECTs, MRIs, and CTs, as detailed in Table S1.

## Synthetic CT generation and SPECT reconstruction

Attenuation- and scatter-corrected SPECT images were generated as follows, with the numbers corresponding to those in Fig. S1:

1. Hermes Hybrid Recon (HERMES Medical Solutions, Stockholm, Sweden) OSEM was used to reconstruct the SPECT scans using UAC from their raw DICOM format and then converted from DICOM to NIfTI. MRI, SPECT, and gCT images were manually aligned to the anterior commissure.
2. A rigid registration was performed to align the gCT with the MRI using normalized mutual information as a similarity measure in Statistical Parametric Mapping (SPM12, Wellcome Trust Centre for Neuroimaging, London, UK). The headrest in the gCT images was removed using the "Remove CT Table" function in 3D Slicer 5.2.2 software.
3. For synthetic CT (sCT) generation, a pretrained model (3D residual U-Net) was used. The preprocessing of MRIs included resampling of RAS+ oriented MRIs to an isotropic voxel size of 1 mm as well as bias field correction. A binary head mask was created from the T1-weighted images using Otsu segmentation to reduce noise outside the head. After applying the head mask, the bias corrected, resampled MRIs were used as input for the neural network to generate sCT. Background noise outside the head was masked out in the sCT.
4. As the gCT field of view (FOV) was typically smaller in the cranio-caudal direction than the sCT, it was adjusted accordingly.
5. After sCT generation, the SPECT and MRI were rigidly registered using normalized mutual information as the similarity measure. The resulting transformation was then applied inversely to the affine matrices of the sCT and gCT (which were already registered to the MRI) to align them with the SPECT.
6. The sCTs and gCTs were resliced to match the corresponding SPECT images using fourth-degree B-spline interpolation in SPM12. They were then converted from NIfTI to DICOM format using an in-house script.
7. For all three datasets NAC, UAC, DL-MRAC and CTAC were performed using Hermes Hybrid Recon. For *prospective validation cohort*, both Monte Carlo-based scatter correction and collimator correction with a Gaussian model were applied. For *external validation cohort 1*, scatter correction was available. However, for *external validation cohort 2*, scatter correction was not possible because the DICOM tag "radius of rotation" was missing and not documented in the PPMI database. Collimator correction could not be applied to either *external validation cohort* because information such as collimator hole diameter and length was not documented in the PPMI database. The conversion of Hounsfield units to attenuation coefficients was performed using a bilinear transformation implemented in the Hermes Software. UAC was applied using an attenuation coefficient of 0.146/cm inside the head, whose outer contour was delineated using an automatic thresholding approach implemented in the Hermes workstation. Monte Carlo based scatter correction was applied based on the attenuation map with 10^5^ simulated photons and two update iterations. The reconstruction process involved 15 iterations and four subsets. All SPECT scans underwent Gaussian post-reconstruction filtering for noise suppression with a full width at half-maximum of 7 mm.

Inference was performed on a Linux workstation with an Intel Xeon Gold 6542Y CPU (86 cores) and 424 GB RAM, using CPU-only execution, requiring approximately 5 minutes per synthetic CT. The framework is compatible with both NVIDIA GPU-based and CPU-only platforms and has also been successfully tested on a low-resource standard laptop (AMD Ryzen 3 CPU, 6 GB RAM), where inference required approximately 40 minutes per synthetic CT.


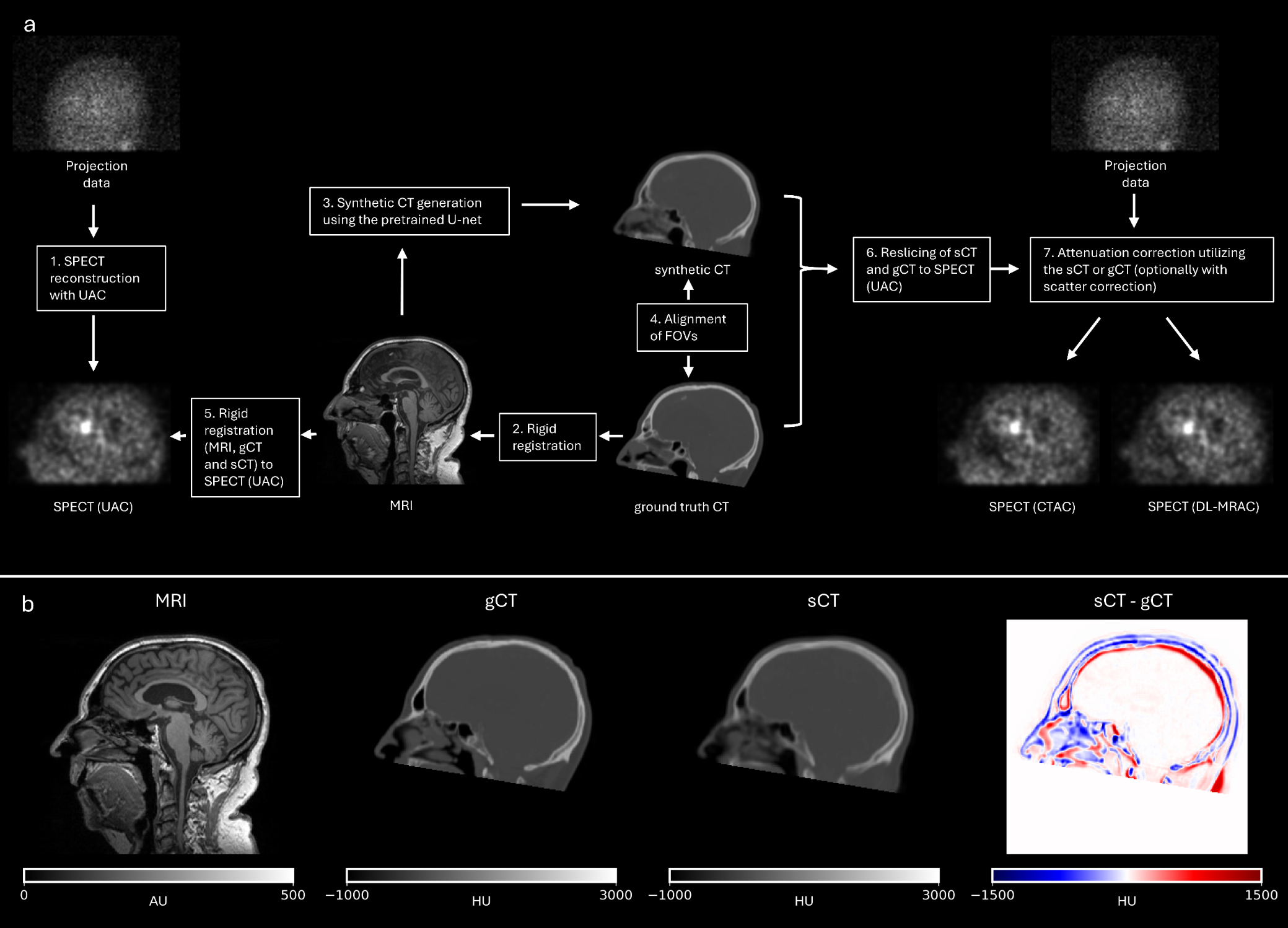


**Fig. S1** Synthetic CT (sCT) generation for SPECT attenuation and scatter correction. a Attenuation and scatter correction of SPECT scans involved the following steps: 1. Reconstruction of the raw SPECT data (projection data) using uniform attenuation correction (UAC). 2. Registration of the ground truth CT (gCT) to the MRI. 3. sCT generation using the bias-corrected, resampled MRI. 4. Alignment of the Field of Views (FOVs) of the sCT and gCT. 5. Registration of the MRI, gCT, and sCT to the SPECT (UAC). 6. Reslicing the registered sCT and gCT (from step 5) to SPECT (previously reconstructed with UAC) 7. Using the gCT and sCT to perform attenuation (and scatter) correction on the projection data. b MRI, gCT, sCT, and the corresponding difference image are shown for a representative subject from the PPMI database. HU: Hounsfield units; AU: arbitrary units. CTAC: ground truth CT-based attenuation correction. DL-MRAC: deep-learning MR-based attenuation correction

# Supplementary Results

## Synthetic CT accuracy

### Prospective validation cohort

Significant differences in the MAE within the head mask and bone window were observed in *prospective validation cohort* depending on the method of bias field correction (N4ITK vs. SPM) and the field of view (enlarged to 208x288x288 voxels vs. the native FOV of 173x230x230 voxels, both with a 1mm isotropic voxel size). The lowest MAE values in both the head mask and bone window were achieved using SPM bias field correction in combination with an enlarged field of view (Fig. S2). This result is contrary to the expectations based on the neural network description, which states that the model was trained on MRI images corrected with N4ITK bias field correction and recommends a maximum matrix size of 256x256x256 (<https://github.com/sitiny/mr-to-pCT>). sCTs generated from MRIs with enlarged FOV and SPM bias field correction were used for attenuation and scatter correction of SPECT data in the *prospective validation cohort*.


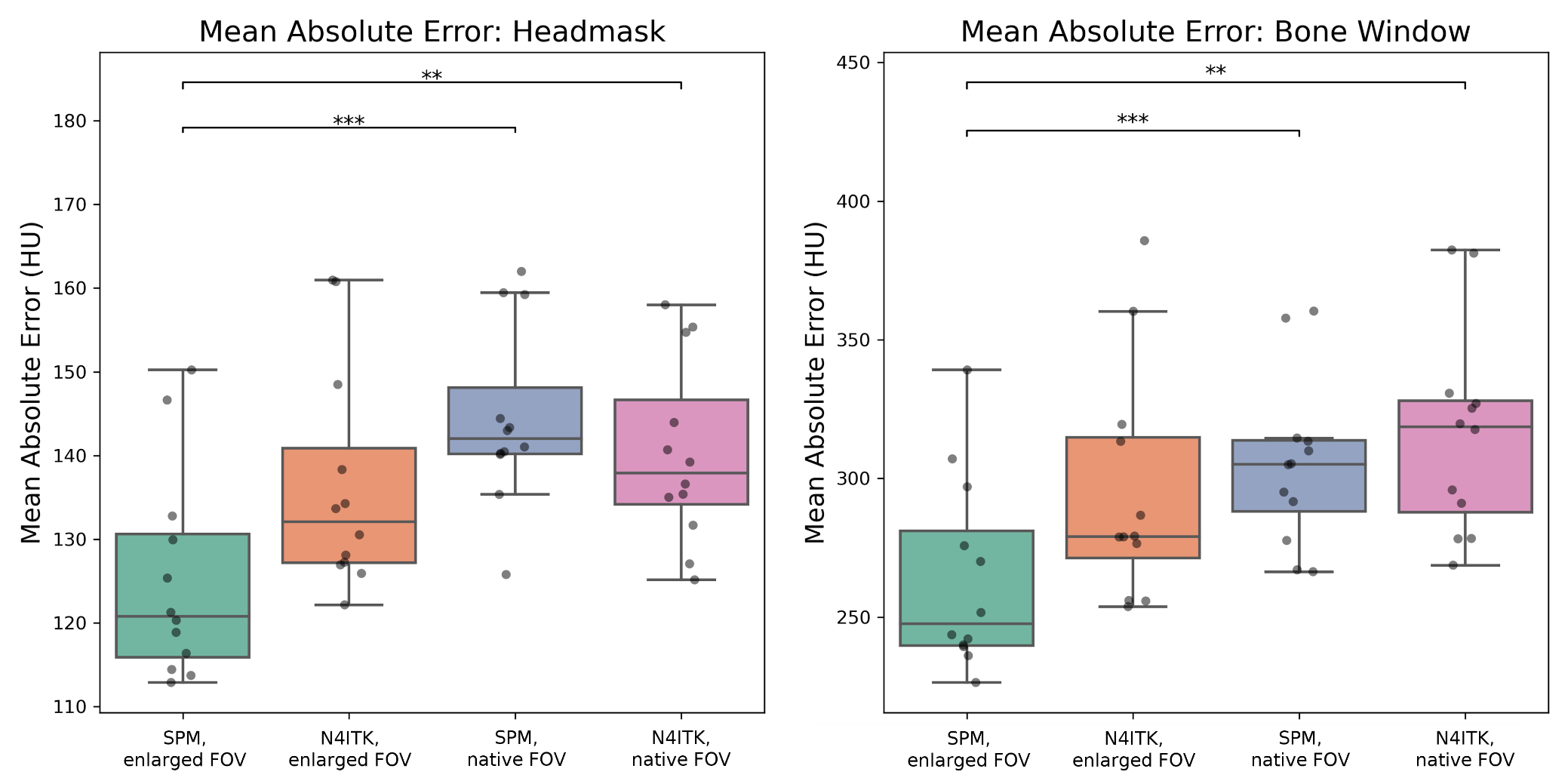


**Fig. S2** Boxplots show the mean absolute error in Hounsfield units (HU) between the gCT and sCT within the head mask and bone window for *prospective validation cohort*. Four different preprocessing methods (SPM or N4ITK bias field correction, combined with either the native or enlarged FOV) were tested. A paired Wilcoxon signed-rank test (*p < 0.05, ** p <0.01, ***p < 0.001) was conducted to assess differences between these methods

### External validation cohort 1

Significant differences in the mean absolute error (MAE) within the head mask and bone window were observed in *external validation cohort 1*, depending on the method of bias field correction (data not shown). However, the combination of the native FOV with N4ITK, as recommended in the neural network's description, did not perform significantly worse than the other methods. sCTs generated from MRIs with the native FOV (176x250x250 voxels, 1mm isotropic voxel size) and N4ITK bias field correction were used for attenuation and scatter correction of SPECT data in *external validation cohort 1*.

### External validation cohort 2

No significant differences in MAE were observed within the head mask or the bone window across the four preprocessing methods (data not shown). sCTs generated from MRIs with the native FOV (176x240x256 voxels, 1mm isotropic voxel size) and N4ITK bias field correction were used for attenuation correction of SPECT data in the *external validation cohort 2*.

## Regional analysis

### External validation cohort 1

The mean bias of the SBR per ROI for the SPECT reconstructed with the sCT in *external validation cohort 1* was within a range of -3.3% to -0.3% with a tendency for underestimation (Fig. S3). There was a significant bias in 43 of 70 regions. UAC resulted in larger errors from -1.6% to 11.4% with a tendency for overestimation and significant bias in 62 regions. SPECT reconstructed with NAC showed the largest error with a range of bias from -22.8% to +7.0% with a tendency for underestimation and significant bias in 57 regions. A summary table for selected ROIs is provided in Table S2.


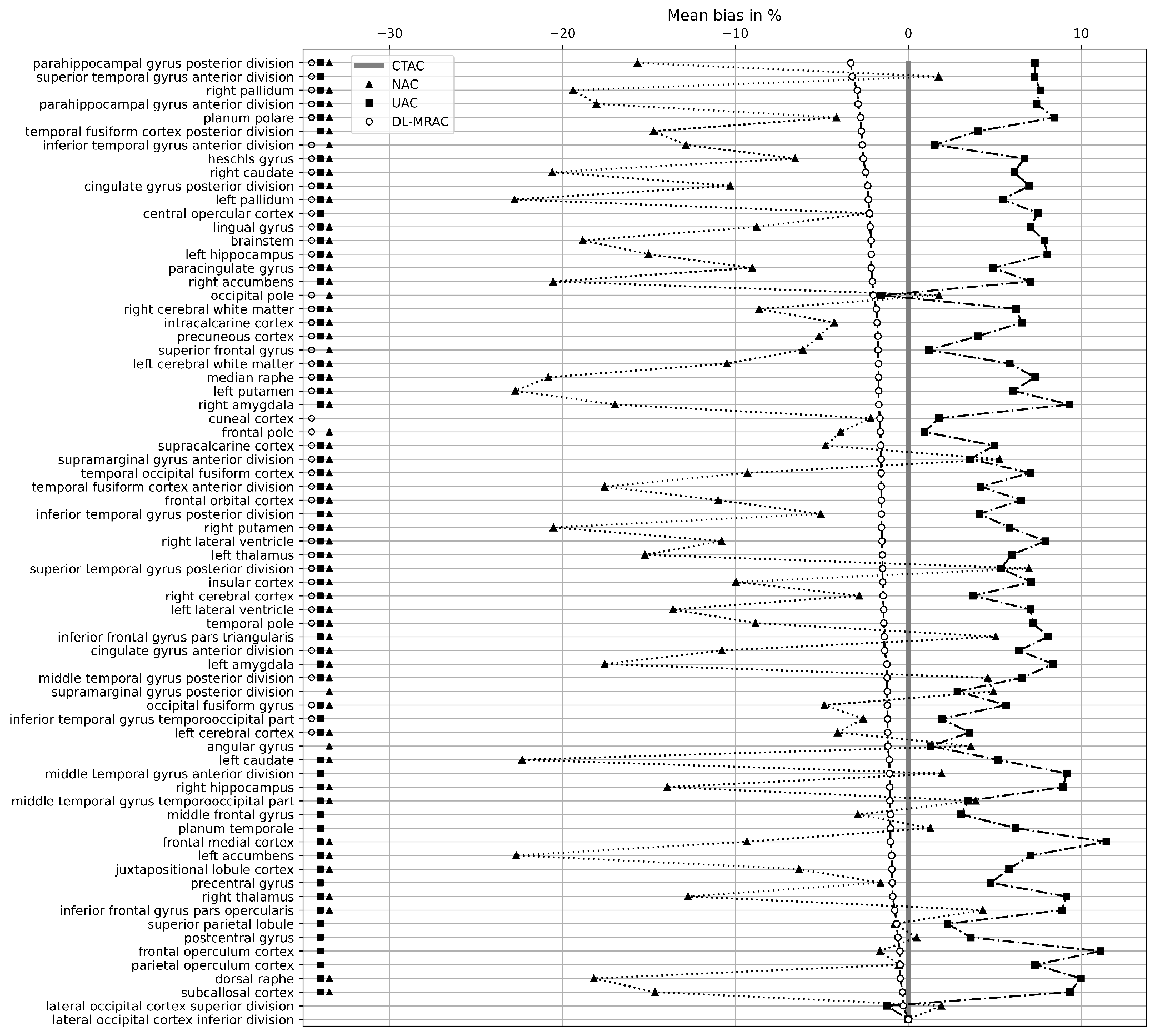


**Fig. S3** External validation cohort 1. Mean SBR bias per ROI and method, sorted by DL-MRAC bias from most negative to most positive. Symbols represent SBR approaches: NAC (triangle), UAC (square), DL-MRAC (circle). Bias in % is represented on the x-axis. Paired Wilcoxon signed rank test at the regional level (p<0.05). On the left side of the figure symbols indicate that the regional SBR of the respective method was significantly different from the ground truth SBR (CTAC)

**Table S2** External validation cohort 1. Mean bias and standard deviation of SBR (%) for the NAC, UAC, and DL-MRAC methods compared to CTAC for the striatum, pallidum, raphe nucleus and brainstem

|  | **NAC** | **UAC** | **DL-MRAC** |
| --- | --- | --- | --- |
| **Caudate L** | -22.34 ± 3.86 * | 5.18 ± 3.08 * | -1.10 ± 1.57 |
| **Caudate R** | -20.59 ± 3.53 * | 6.13 ± 2.95 * | -2.46 ± 1.04 * |
| **Putamen L** | -22.72 ± 3.72 * | 6.08 ± 3.00 * | -1.71 ± 1.80 * |
| **Putamen R** | -20.52 ± 4.27 * | 5.86 ± 2.65 * | -1.55 ± 1.57 * |
| **Pallidum L** | -22.78 ± 3.98 * | 5.46 ± 4.03 * | -2.31 ± 1.61 * |
| **Pallidum R** | -19.38 ± 4.87 * | 7.63 ± 4.45 * | -2.94 ± 2.61 * |
| **Dorsal Raphe** | -18.18 ± 3.44 * | 9.98 ± 4.88 * | -0.47 ± 3.73 |
| **Median Raphe** | -20.82 ± 2.53 * | 7.34 ± 4.51 * | -1.72 ± 1.87 * |
| **Brainstem** | -18.83 ± 2.21 * | 7.86 ± 3.11 * | -2.15 ± 1.02* |

*p<0.05 Wilcoxon signed rank test. R, right, L, left

### External validation cohort 2

The mean bias of SBR per ROI for the SPECT reconstructed with DL-MRAC was between -1.7% and +2.6%. There was a significant bias in 8 of 70 regions (Fig. S4). UAC resulted in larger errors from ‑8.5% to +16.9% with a tendency for overestimation and a significant bias in 61 regions. NAC showed the largest error with a range of bias from -23.2% to +5.4% with a tendency for underestimation and a significant bias in 61 regions. A clearer presentation for selected ROIs is provided in Table S3.


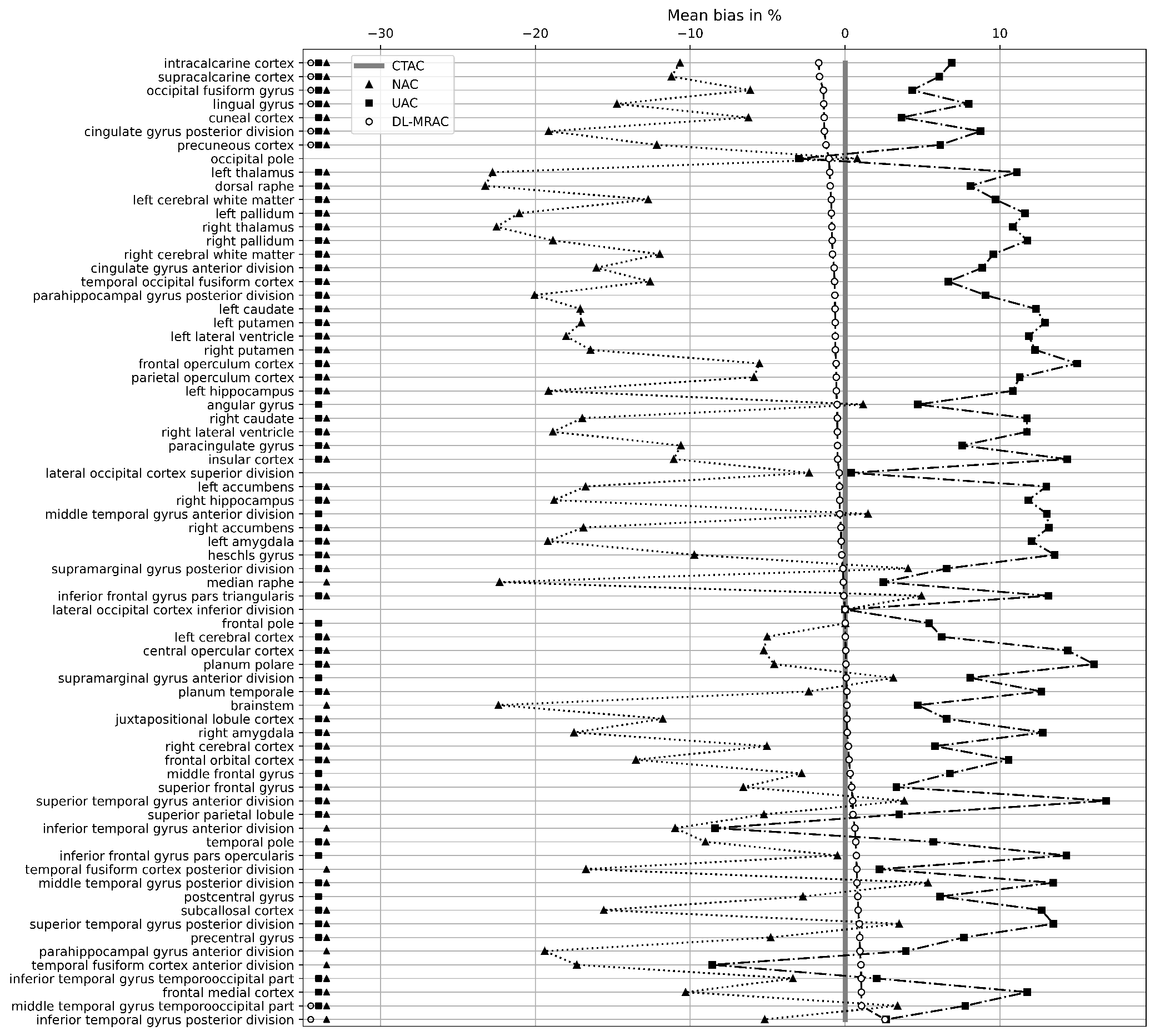


**Fig. S4** External validation cohort 2. Mean SBR bias per ROI and method, sorted by DL-MRAC bias from most negative to most positive. Symbols represent SBR approaches: NAC (triangle), UAC (square), DL-MRAC (circle). Bias in % is represented on the x-axis. Paired Wilcoxon signed rank test at the regional level (p<0.05). On the left side of the figure symbols indicate that the regional SBR of the respective method was significantly different from the ground truth SBR (CTAC)

**Table S3** External validation cohort 2. Mean bias and standard deviation of SBR (%) for the NAC, UAC, and DL-MRAC methods compared to CTAC for the striatum, pallidum, raphe nucleus and brainstem

|  | **NAC** | **UAC** | **DL-MRAC** |
| --- | --- | --- | --- |
| **Caudate L** | -17.10 ± 2.84 * | 12.32 ± 2.28 * | -0.64 ± 1.71 |
| **Caudate R** | -16.97 ± 2.23 * | 11.74 ± 1.55 * | -0.50 ± 1.67 |
| **Putamen L** | -17.03 ± 2.20 * | 12.90 ± 2.54 * | -0.64 ± 1.51 |
| **Putamen R** | -16.44 ± 2.84 * | 12.27 ± 1.29 * | -0.63 ± 1.47 |
| **Pallidum L** | -21.04 ± 1.31 * | 11.62 ± 2.55 * | -0.89 ± 1.43 |
| **Pallidum R** | -18.86 ± 2.82 * | 11.77 ± 1.66 * | -0.83 ± 1.37 |
| **Dorsal Raphe** | -23.24 ± 2.45 * | 8.09 ± 3.20 * | -0.96 ± 1.31 |
| **Median Raphe** | -22.31 ± 3.12 * | 2.47 ± 8.52 | -0.11 ± 1.45 |
| **Brainstem** | -22.39 ± 1.80 * | 4.70 ± 6.54 | 0.12 ± 1.28 |

*p<0.05 Wilcoxon signed rank test. R, right, L left


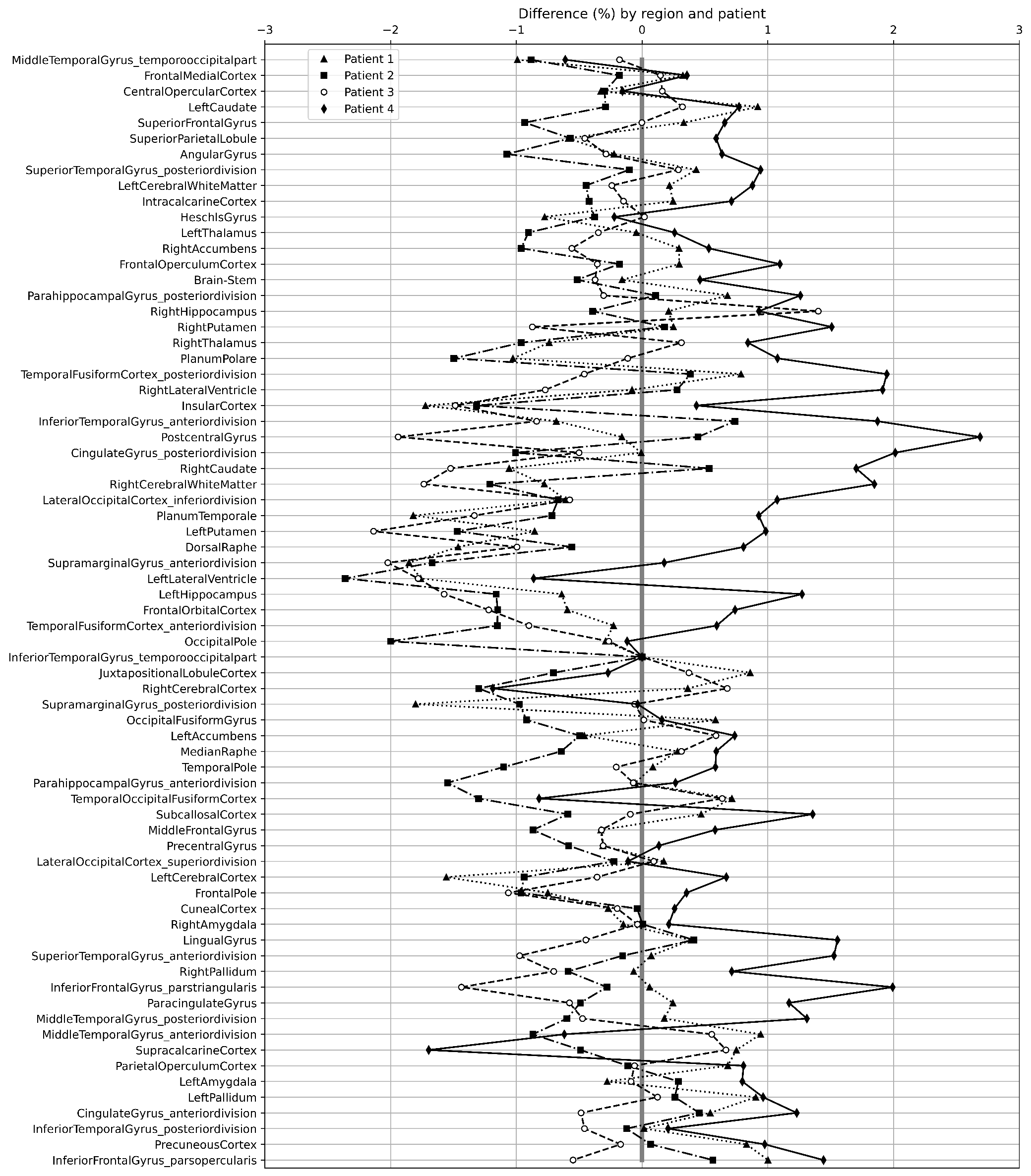


**Fig. S5** External validation cohort 2. Test-retest analysis for DL-MRAC. Mean SBR bias per ROI for each of the four patients with a repeat MRI available. Bias (%) is shown as the difference between SPECT attenuation-corrected using the synthetic CT generated from the baseline MRI and that generated from the repeat MRI

**Table S4** External validation cohort 2. Test-retest analysis for DL-MRAC. Mean SBR bias per ROI (%) for each of the four patients with a repeat MRI available for the striatum, pallidum, raphe nucleus and brainstem

|  | **Patient 1** | **Patient 2** | **Patient 3** | **Patient 4** |
| --- | --- | --- | --- | --- |
| **Caudate L** | 0.43 | -0.10 | 0.29 | 0.94 |
| **Caudate R** | 0.90 | 0.26 | 0.12 | 0.96 |
| **Putamen L** | 0.22 | -0.45 | -0.24 | 0.88 |
| **Putamen R** | 0.54 | 0.46 | -0.49 | 1.23 |
| **Pallidum L** | 0.25 | -0.42 | -0.15 | 0.71 |
| **Pallidum R** | 0.68 | 0.11 | -0.31 | 1.26 |
| **Dorsal Raphe** | -0.33 | -0.30 | 0.16 | -0.16 |
| **Median Raphe** | -0.99 | -0.88 | -0.18 | -0.61 |
| **Brainstem** | -0.77 | -0.38 | 0.02 | -0.22 |

## Voxel-wise analysis


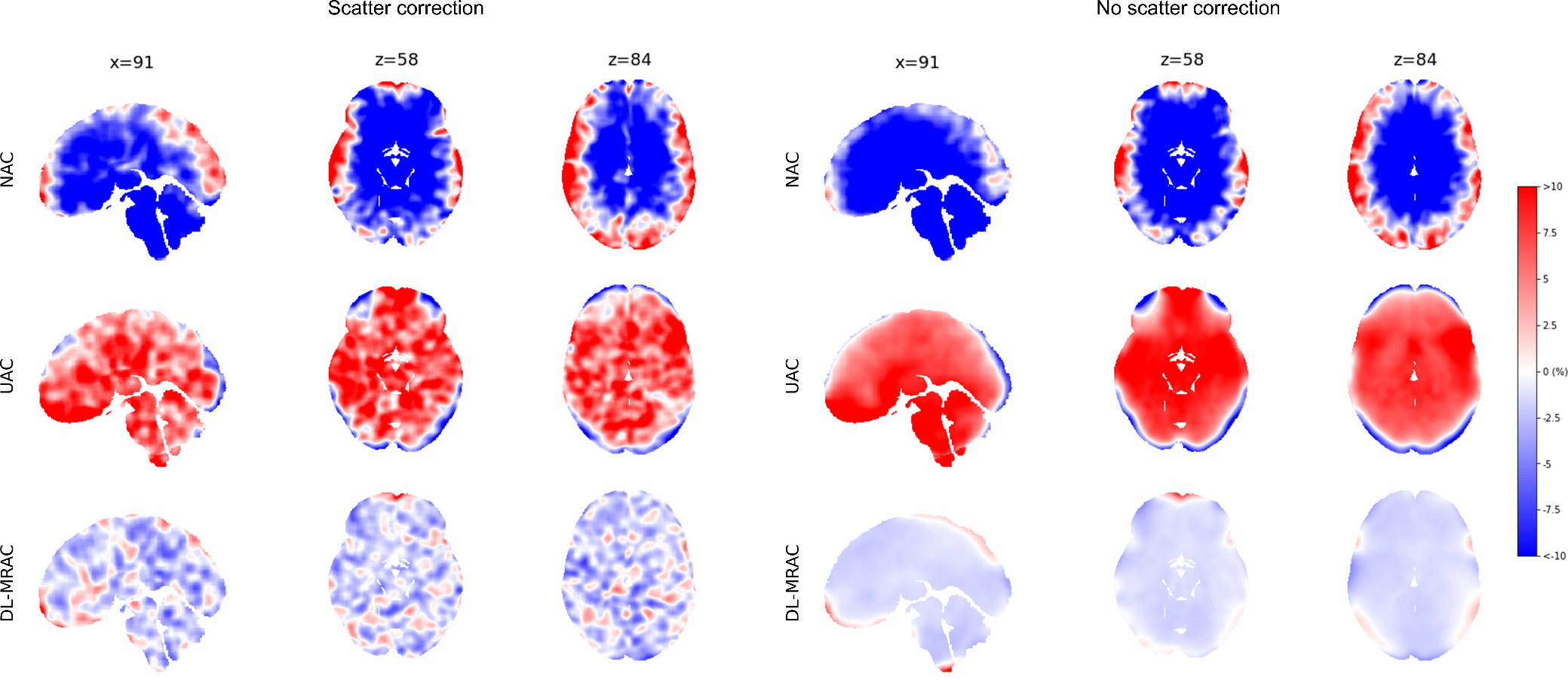


**Fig. S6** External validation cohort 1. Direct comparison of the mean voxel-wise SBR bias (in %) in MNI152 space for scatter correction (left) and the absence of scatter correction (right). Z=58 goes through the midbrain, z=84 through the caput of the caudate. The colour scale was thresholded at -10% and +10% to improve the visualization of local errors


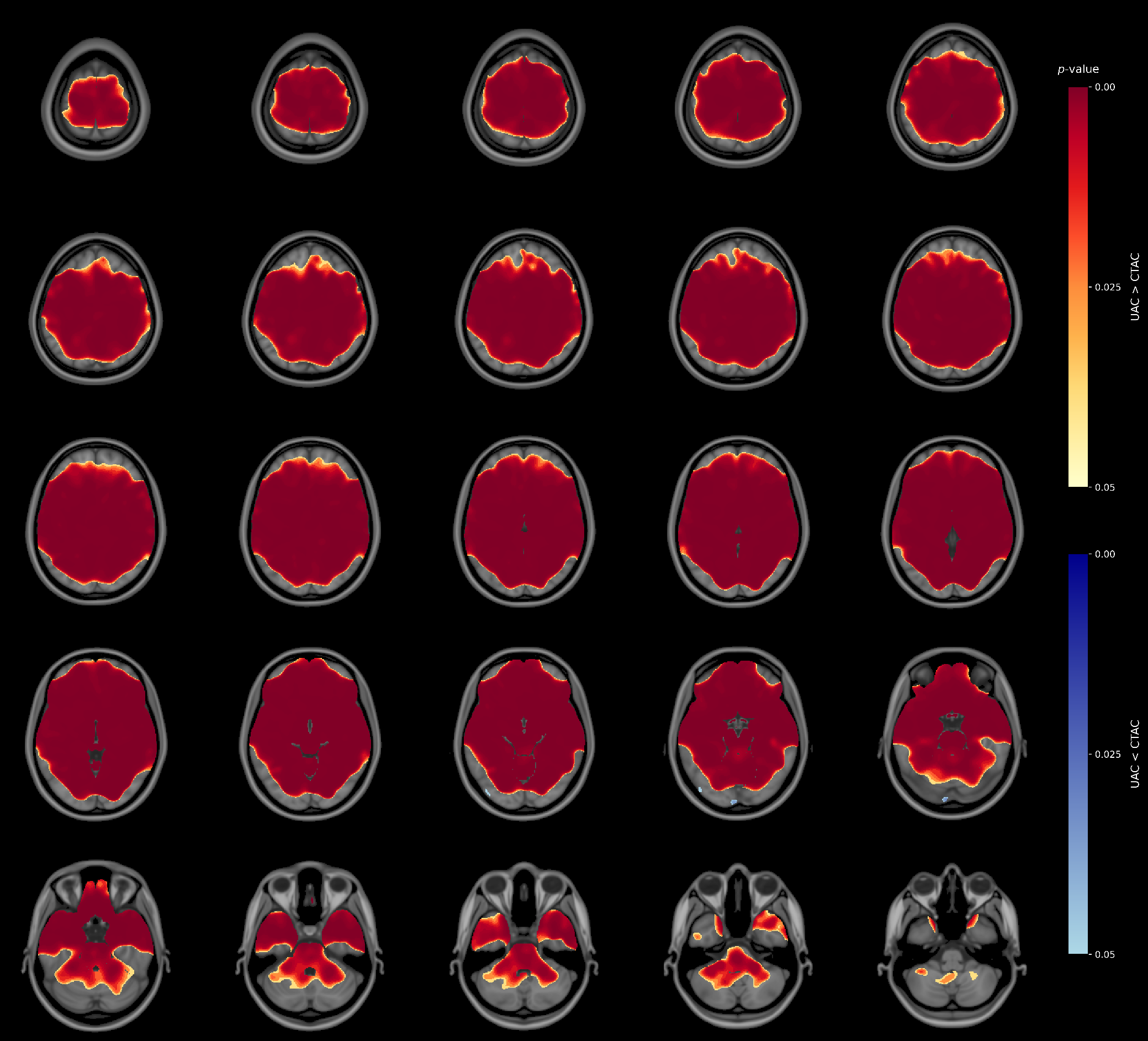


**Fig. S7** Voxel-wise paired testing of UAC versus CTAC in the prospective validation cohort. Significant clusters of overestimation (UAC > CTAC) are shown in red, whereas significant clusters of underestimation (UAC < CTAC) are shown in blue. Clusters not reaching statistical significance (p < 0.05) are not displayed.


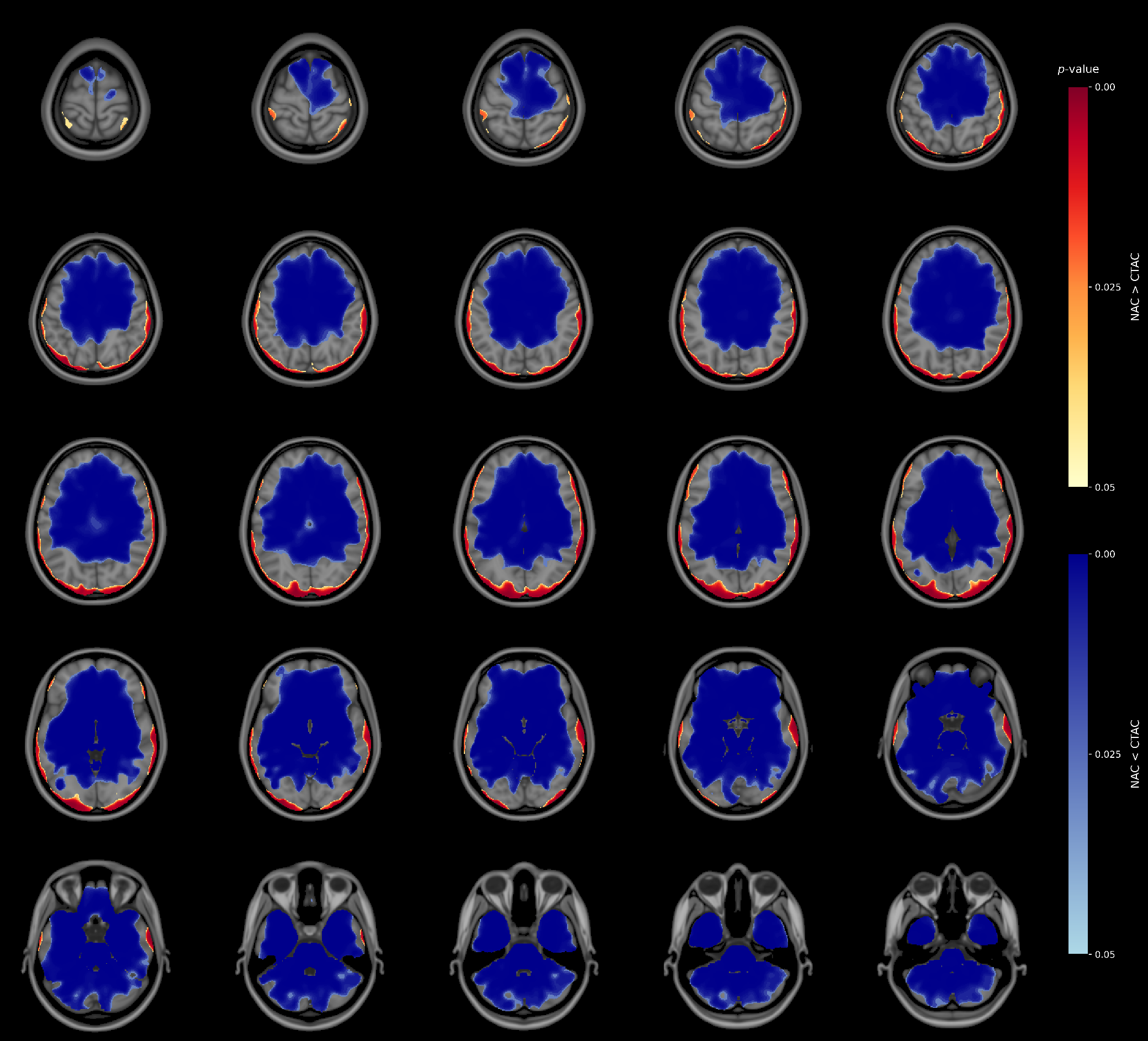


**Fig. S8** Voxel-wise paired testing of NAC versus CTAC in the prospective validation cohort. Significant clusters of overestimation (NAC > CTAC) are shown in red, whereas significant clusters of underestimation (NAC < CTAC) are shown in blue. Clusters not reaching statistical significance (p < 0.05) are not displayed.


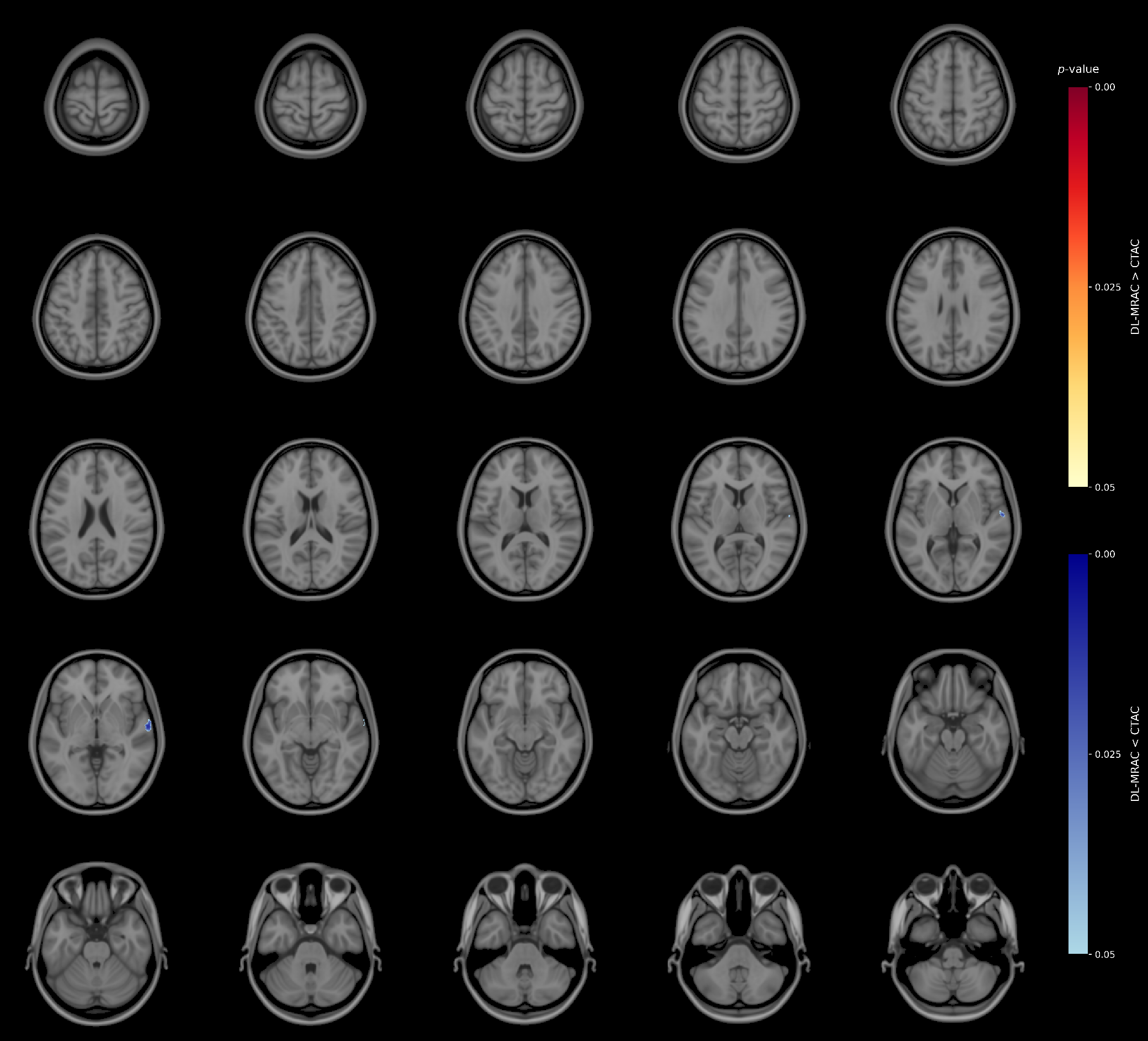


**Fig. S9** Voxel-wise paired testing of DL-MRAC versus CTAC in the external validation cohort 1. Significant clusters of overestimation (DL-MRAC > CTAC) are shown in red, whereas significant clusters of underestimation (DL-MRAC < CTAC) are shown in blue. Clusters not reaching statistical significance (p < 0.05) are not displayed.


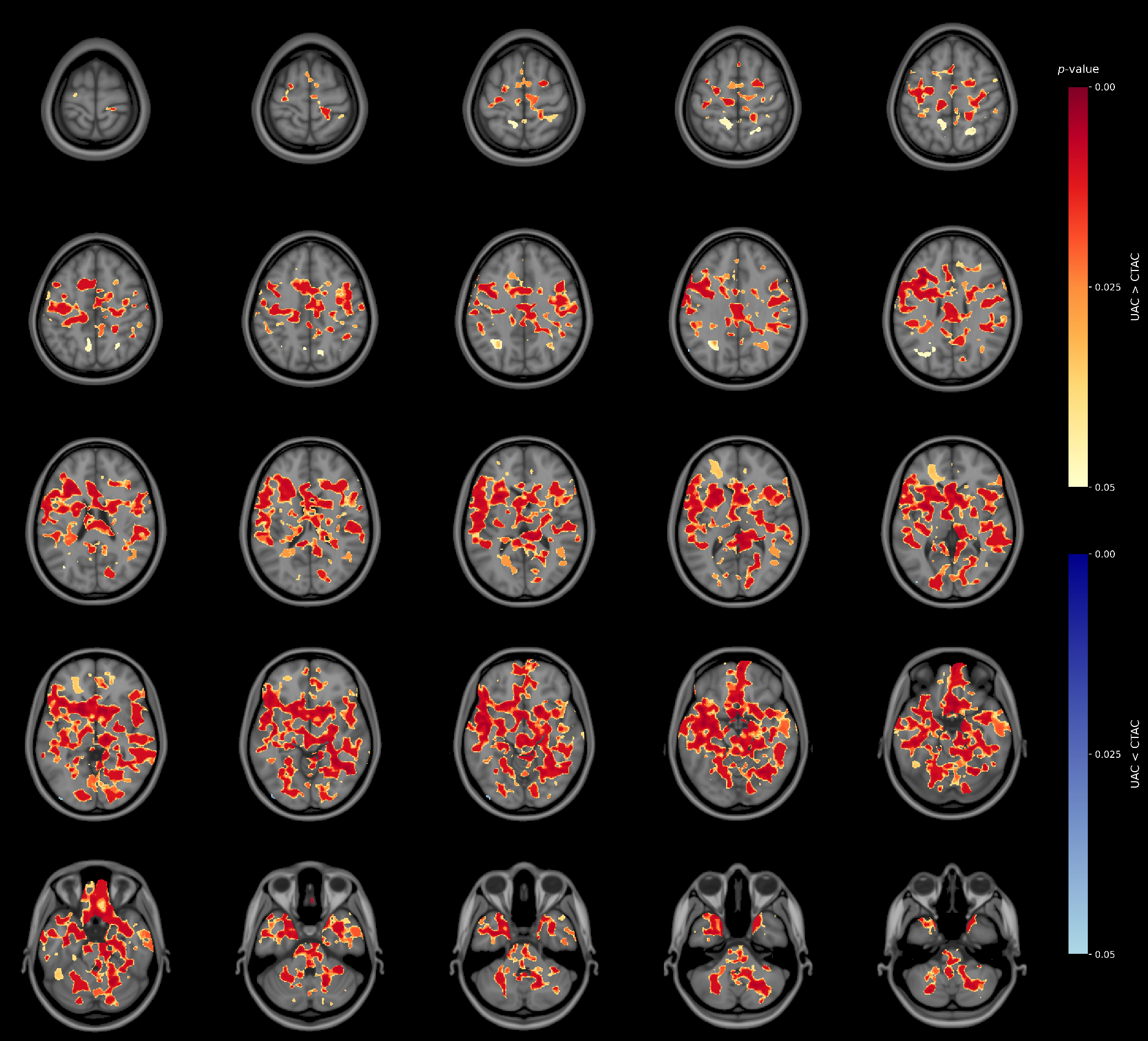


**Fig. S10** Voxel-wise paired testing of UAC versus CTAC in the external validation cohort 1. Significant clusters of overestimation (UAC > CTAC) are shown in red, whereas significant clusters of underestimation (UAC < CTAC) are shown in blue. Clusters not reaching statistical significance (p < 0.05) are not displayed.


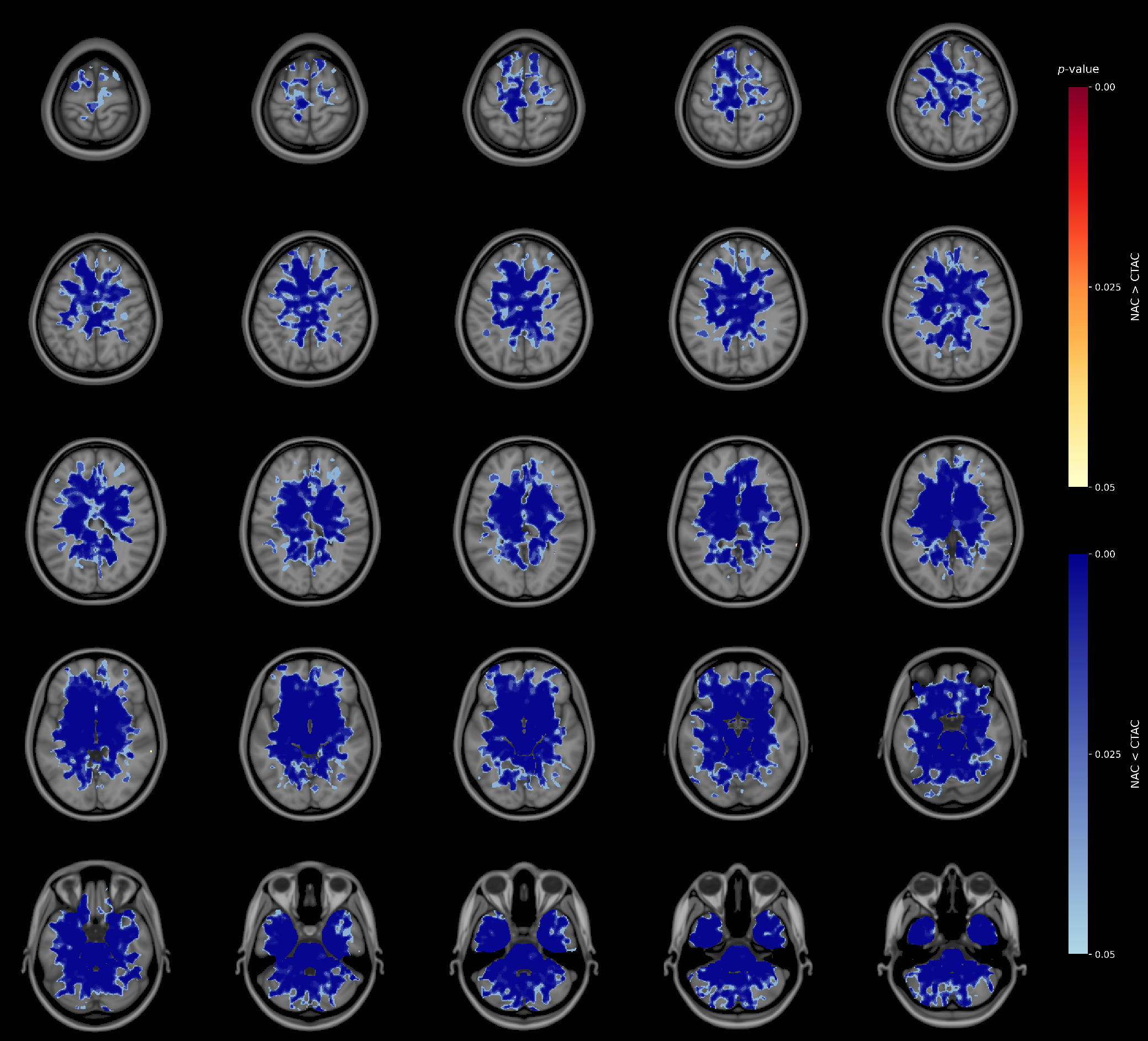


**Fig. S11** Voxel-wise paired testing of NAC versus CTAC in the external validation cohort 1. Significant clusters of overestimation (NAC > CTAC) are shown in red, whereas significant clusters of underestimation (NAC < CTAC) are shown in blue. Clusters not reaching statistical significance (p < 0.05) are not displayed.


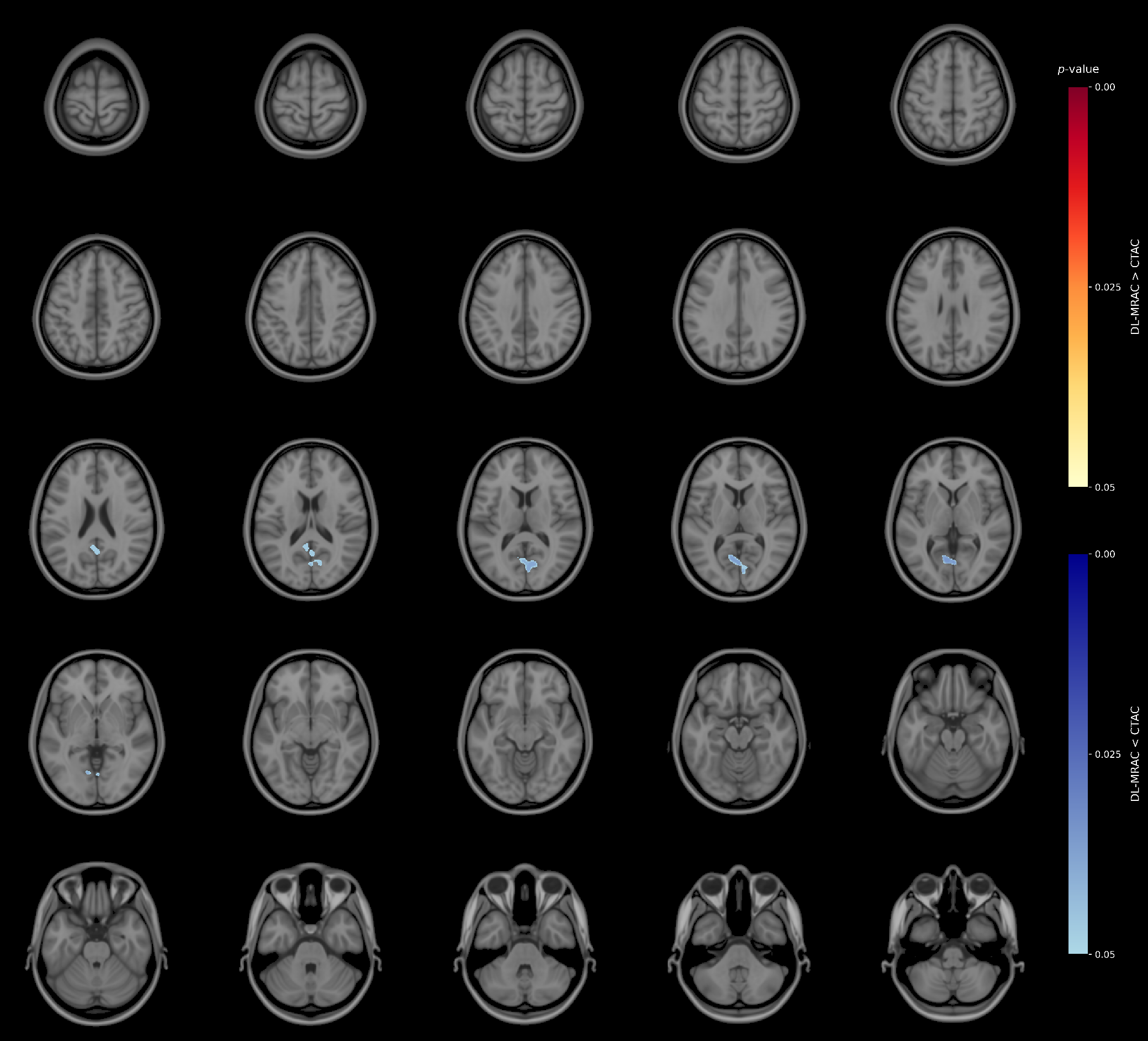


**Fig. S12** Voxel-wise paired testing of DL-MRAC versus CTAC in the external validation cohort 2. Significant clusters of overestimation (DL-MRAC > CTAC) are shown in red, whereas significant clusters of underestimation (DL-MRAC < CTAC) are shown in blue. Clusters not reaching statistical significance (p < 0.05) are not displayed.


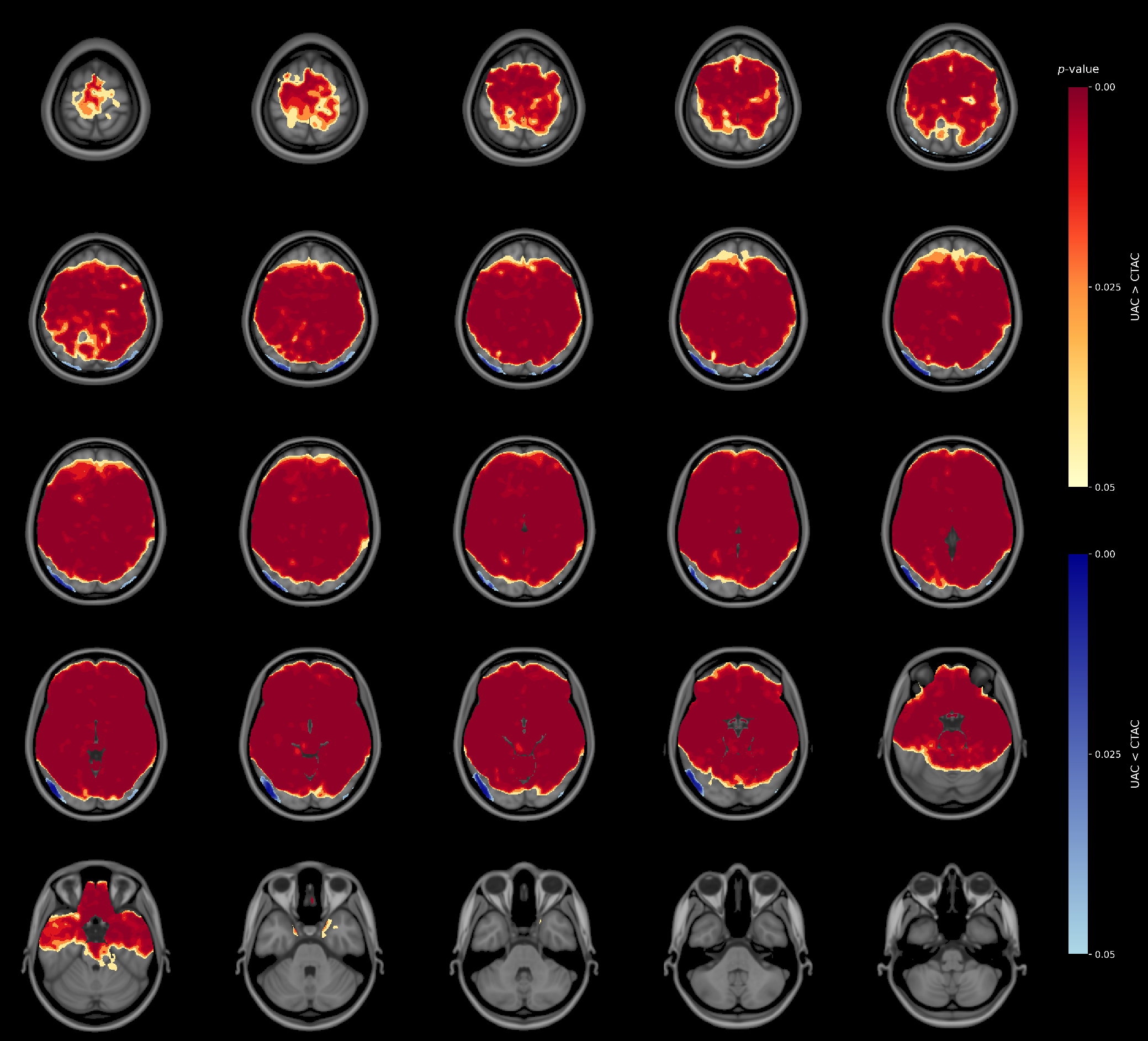


**Fig. S13** Voxel-wise paired testing of UAC versus CTAC in the external validation cohort 2. Significant clusters of overestimation (UAC > CTAC) are shown in red, whereas significant clusters of underestimation (UAC < CTAC) are shown in blue. Clusters not reaching statistical significance (p < 0.05) are not displayed.


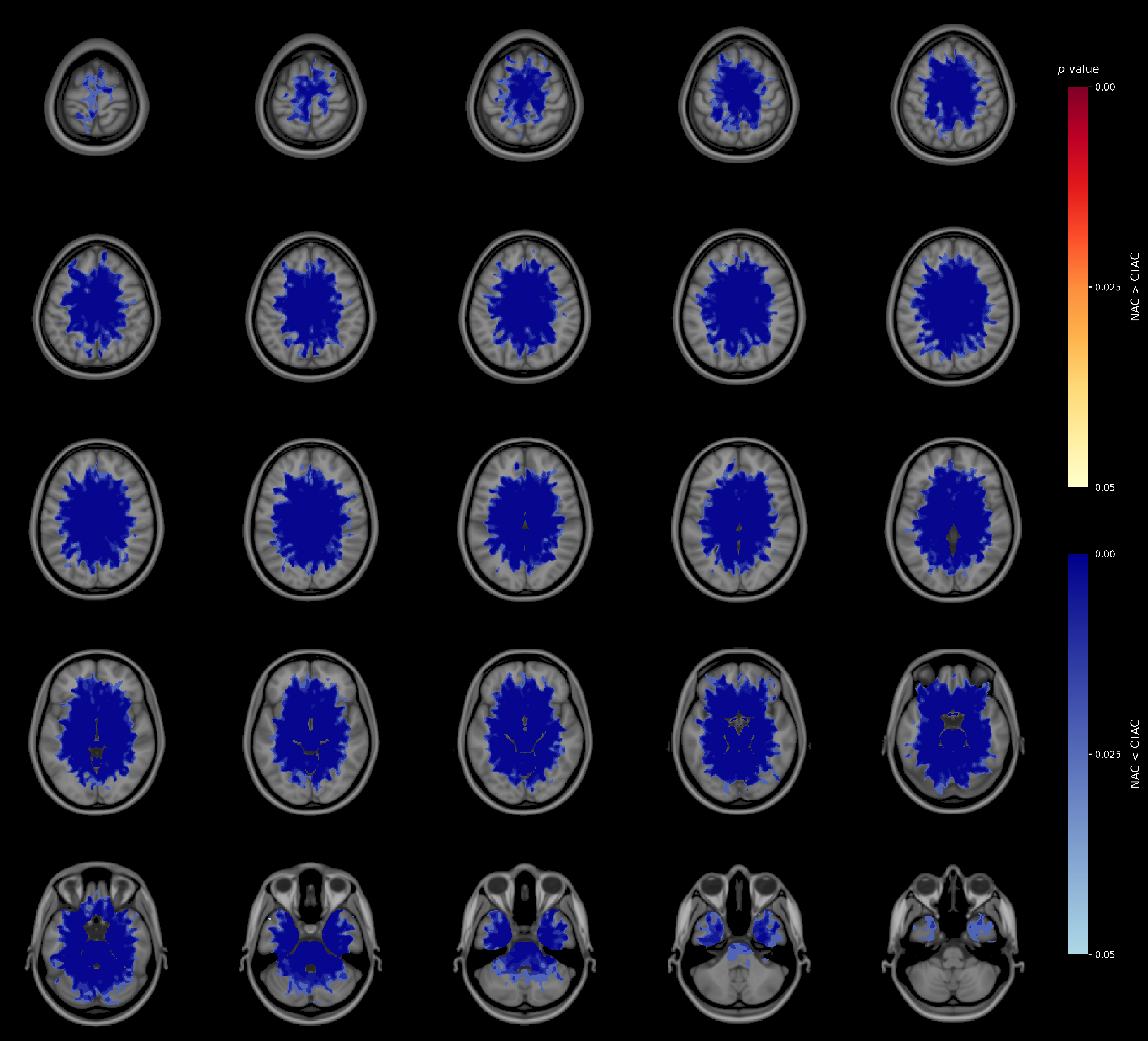


**Fig. S14** Voxel-wise paired testing of NAC versus CTAC in the external validation cohort 2. Significant clusters of overestimation (NAC > CTAC) are shown in red, whereas significant clusters of underestimation (NAC < CTAC) are shown in blue. Clusters not reaching statistical significance (p < 0.05) are not displayed.
